# Neuroprotective Effects of Coffee Bioactive Compounds: A Review

**DOI:** 10.3390/ijms22010107

**Published:** 2020-12-24

**Authors:** Katarzyna Socała, Aleksandra Szopa, Anna Serefko, Ewa Poleszak, Piotr Wlaź

**Affiliations:** 1 Department of Animal Physiology and Pharmacology, Institute of Biological Sciences, Maria Curie-Skłodowska University, Akademicka 19, 20-033 Lublin, Poland; piotr.wlaz@poczta.umcs.lublin.pl; 2 Laboratory of Preclinical Testing, Chair and Department of Applied and Social Pharmacy, Medical University of Lublin, Chodźki 1, 20-093 Lublin, Poland; aleksandra.szopa@umlub.pl (A.S.); anna.serefko@umlub.pl (A.S.); ewa.poleszak@umlub.pl (E.P.)

**Keywords:** coffee consumption, caffeine, chlorogenic acid, caffeic acid, trigonelline, neuroprotection, Alzheimer’s disease, Parkinson’s disease, stroke

## Abstract

Coffee is one of the most widely consumed beverages worldwide. It is usually identified as a stimulant because of a high content of caffeine. However, caffeine is not the only coffee bioactive component. The coffee beverage is in fact a mixture of a number of bioactive compounds such as polyphenols, especially chlorogenic acids (in green beans) and caffeic acid (in roasted coffee beans), alkaloids (caffeine and trigonelline), and the diterpenes (cafestol and kahweol). Extensive research shows that coffee consumption appears to have beneficial effects on human health. Regular coffee intake may protect from many chronic disorders, including cardiovascular disease, type 2 diabetes, obesity, and some types of cancer. Importantly, coffee consumption seems to be also correlated with a decreased risk of developing some neurodegenerative conditions such as Alzheimer’s disease, Parkinson’s disease, and dementia. Regular coffee intake may also reduce the risk of stroke. The mechanism underlying these effects is, however, still poorly understood. This review summarizes the current knowledge on the neuroprotective potential of the main bioactive coffee components, i.e., caffeine, chlorogenic acid, caffeic acid, trigonelline, kahweol, and cafestol. Data from both in vitro and in vivo preclinical experiments, including their potential therapeutic applications, are reviewed and discussed. Epidemiological studies and clinical reports on this matter are also described. Moreover, potential molecular mechanism(s) by which coffee bioactive components may provide neuroprotection are reviewed.

## 1. Introduction

The genus *Coffea* L. (family: *Rubiaceae*, subfamily: *Ixoroideae*, tribe: *Coffeeae*) includes at least 125 species which naturally occur in Tropical and East Africa, Tropical Asia, and Australia and also in the Comoros, Madagascar, and the Mascarenes [1]. Only three of these species are used in the commercial coffee production, i.e., *Coffea arabica L*. (Arabica coffee), *Coffea canephora* Pierre ex A. Froehner (Robusta coffee), and *Coffea liberica* Hiern (Excelsa coffee) [2,3,4,5,6].

Coffee beans are obtained from the tart red fruit of the evergreen coffee tree. They are used primarily in the food industry but also in cosmetology and medicine. Nowadays, coffee is considered to be one of the most highly popular and widely consumed pharmacologically active universal beverages [7,8], and its drinking has become a regular part of daily life [9]. It is estimated that in 2019/2020 world coffee consumption amounted to about 10.1 million kg [10]. Most of all coffee is used due to its psychostimulating effect, taste and aroma as well as health-promoting properties [11].

The health effects of coffee consumption have been investigated in numerous research [12,13,14,15,16,17,18,19]. The outcomes from many of these studies showed the positive impact of coffee intake on various aspects of health, e.g., coffee possesses anti-oxidant (especially the medium-roasted coffee) [20] and anti-inflammatory properties [15] and limits the overall risk of stroke and coronary heart disease [21,22,23], cancer [22,24,25], mortality associated with cardiovascular disease [22,26], Parkinson’s [22,27,28] and Alzheimer’s disease and other neurodegenerative disorders [29,30], depression and suicide [31,32], liver damage particularly in patients at high risk for liver disease, such as cirrhosis, hepatocellular carcinoma and hepatic injury [22,23,33], and developing type 2 diabetes [7,19,22,23]. However, excessive coffee drinkers also experience negative effects of its use, e.g., caffeine raises concentration of total cholesterol and lowers high density lipoprotein in serum [34] and causes cardiovascular problems, including increased blood pressure, tachycardia, and arrhythmia [21,23,24].

**Figure 1:**
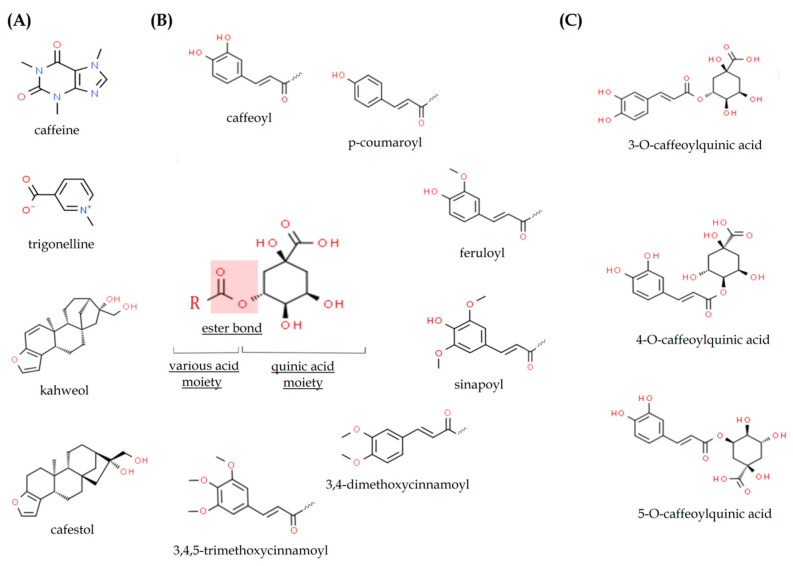
Structures of the most important bioactive compounds in coffee. (**A**) structures of key compounds not belonging to chlorogenic acids, (**B**) general structure of chlorogenic acids and the most important groups found in chlorogenic acids from coffee beans, (**C**) structures of caffeoylquinic acids found in coffee beans.

**Figure 2:**
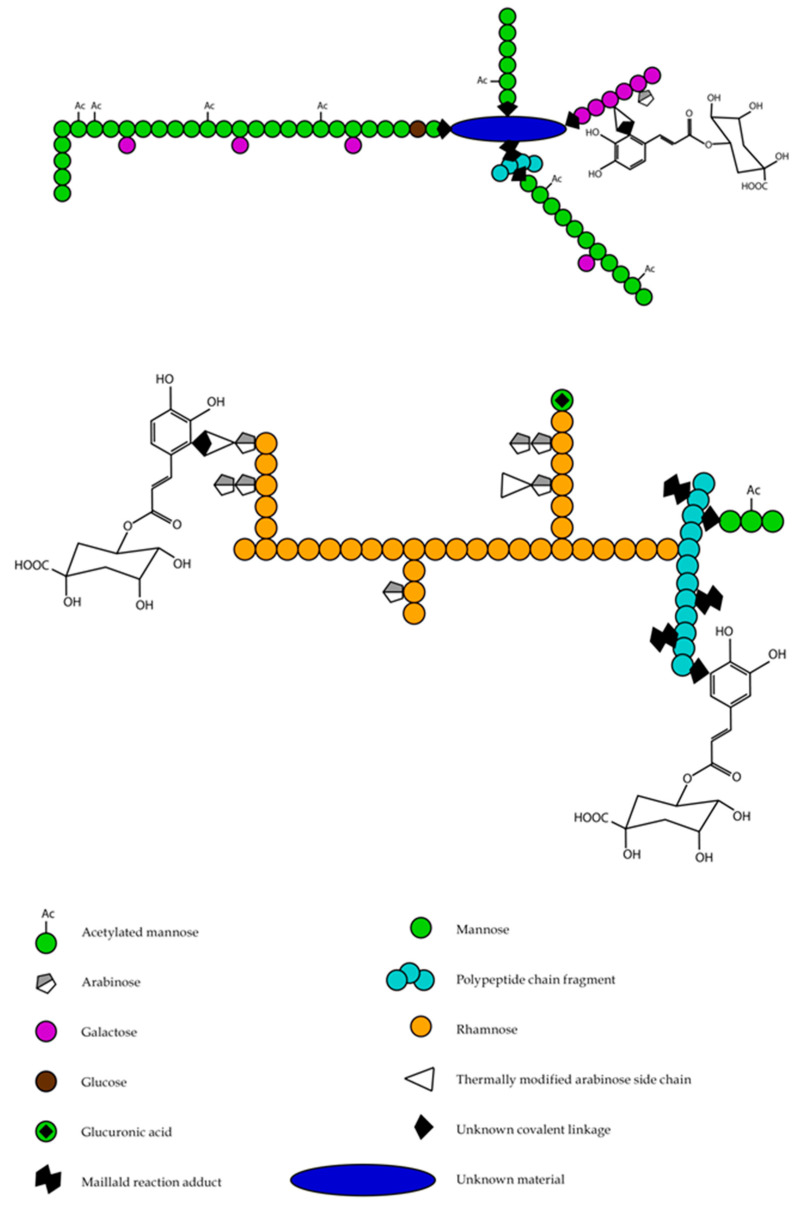
Examples of the structure of coffee melanoidins [38,39].

Such multidirectional effects of coffee on the human health and body are due to the fact that it is a complex mixture of bioactive ingredients and both nutrients and non-nutrients which act together [35]. The composition of these elements in coffee beans differs and depends on (1) species of coffee; (2) conditions of roasting of the coffee beans, including temperature, time, and speed of this process; (3) coffee brewing conditions, i.e., the brewing method, coffee/water ratio, temperature of water, size of coffee grind, and duration of this process [35,36,37]. The most important bioactive compounds in coffee that might serve as physiologically effective agents include caffeine, chlorogenic acids, cafestol and kahweol, trigonelline (Figure 1), and melanoidins (Figure 2) [17,38,39]. The detailed chemical composition and content of active, nutritional, and mineral substances in green and roasted coffee beans and coffee beverage or brew are given in Table 1 and Table 2, respectively.

## 2. Bioavailability and Pharmacokinetics of Coffee Bioactive Compounds

### 2.1. Caffeine

Caffeine is rapidly absorbed–primarily from the small intestine, but also partially from the stomach. According to Arnoud [40], the peak plasma concentration of caffeine (4–5 mg/kg) is observed within 30–120 min after administration with half-lives usually ranged between 2.5 and 5 h. It seems that caffeine absorption is not influenced by age, gender, genetics, undergoing disease, concomitant drugs, or stimulants such as alcohol and nicotine. Caffeine is distributed to all body fluids (including plasma, saliva, bile, cerebrospinal fluid, breast milk, semen, and umbilical cord blood) and to all tissue organs. Due to its lipophilic properties, it crosses cellular membranes easily, including the placental barrier and the blood–brain barrier. Caffeine’s plasma protein binding is limited since its blood/plasma ratio is almost equal to 1. Physiologically no long-term accumulation of this compound or its metabolites is observed [41,42,43,44].

In humans, pharmacokinetics of caffeine also is not affected by the hepatic first-pass effect, and its elimination is regarded as a first-order process described by a one-compartment open model system within the intake range of 2–10 mg/kg [45,46,47]. Caffeine pharmacokinetics may be affected by food and gastric emptying [48], fluid intake [49], and genetic and environmental factors [50], but not by chronovariation [51] or gender [52]. The major caffeine metabolites are paraxanthine, theobromine, and theophylline. All of them are biologically active. Several cytochrome P450 (CYP) isoforms are implicated in caffeine demethylation and C8 hydroxylation (i.e., CYP1A2, CYP1A1, CYP2E1, CYP2D6-Met, and CYP3A), but liver CYP1A2 is mainly responsible for caffeine clearance. Therefore, disturbances of CYP1A2 functioning due to for example genetic polymorphisms or exposure to its inducers significantly influence caffeine metabolism [53,54]. CYP1A2-related modifications in caffeine metabolism were observed during pregnancy or in smoking women taking oral contraceptives [55]. Pharmacokinetics of this methylxanthine may also be affected by genetic determinants [56], specific diet (grapefruit juice, quercetin, brassica vegetables, apiaceous vegetables, large quantities of vitamin C, curcumin, turmeric) [57,58,59,60,61] and lifestyle (i.e., smoking) [62], environmental factors, diseases (particularly liver conditions) [63,64] or concurrent drugs (i.e., clozapine, rofecoxib, quinolones, calcium antagonists, and antiarrhythmics) [65,66,67,68]. However, at least in humans, aging does not impact caffeine metabolism [69,70]. Renal excretion of caffeine dominates in both animals and humans, and ca. 70% of the received caffeine dose is recovered in urine. Approximately 0.5–2% of caffeine is excreted in an unchanged form [71].

### 2.2. Chlorogenic Acids

Chlorogenic acids are a family of esters formed between *trans*-cinnamic acids and quinic acid. They can be divided into three main groups: caffeoylquinic acids, dicaffeoylquinic acids, and feruloylquinic acids. The most abundant chlorogenic acid in coffee beans and other plant sources is 5-O-caffeoylquinic acid, also called chlorogenic acid or wrongly 3-O-caffeoylquinic acid. This is due to the fact that the term “chlorogenic acid” originally referred to 3-O-caffeoylquinic acid. In 1976, the International Union of Pure and Applied Chemistry reversed the order of numbering of atoms on the quinic acid ring and the name for 3-O-caffeoylquinic acid is really 5-O-caffeoylquinic acid [72,73].

In humans, chlorogenic acids are either absorbed untransformed in the stomach and/or duodenum, or absorbed in the stomach and/or small intestine and further metabolized, or subjected to metabolism mediated by gut microbiota with subsequent absorption of catabolites that are not further metabolized or subjected to metabolism mediated by gut microbiota with subsequent absorption of catabolites that are further metabolized (i.e., by reduction, demethylation, dehydroxylation, isomerization, and others) [74]. About 1/3 of consumed chlorogenic acids are absorbed in the small intestine [75,76] and about 2/3 of consumed chlorogenic acids is absorbed in the large intestine. According to the literature data [77,78,79], absorption of the chlorogenic acids in the stomach and in the intestine occurs mainly by passive diffusion with contribution of the active/facilitated transport for several compounds. Though individual differences are noted [75,80], C_max_ of chlorogenic acids that are metabolized in the stomach and/or small intestine is detected relatively quickly, i.e., within 1–2 h. Fatty or sweet food as well as pectins due to diminished rate of gastric emptying can delay detection of their t_max_ values which may result in prolonged plasma clearance. C_max_ of chlorogenic acids that require gut microbiota-related metabolism occurs much later, i.e., within ≥5 h. Absorption of the chlorogenic acids metabolized by gut microbiota is only observed in patients with intact colon. Metabolites obtained after absorption in the stomach and/or small intestine are cleared from plasma within 5–6 h, but colon-associated metabolites may be detectable in plasma after 24 h. Some metabolites are biphasic and show both an early and a late t_max_ values. They also can be still present in plasma after 24 h [81]. Apart from the primary metabolites, chlorogenic acids are also detected in plasma in conjugated forms. Usually chlorogenic acids from green and roasted coffee are absorbed in ca. 33% [82,83,84], but in ileostomized patients the absorption range is between 8% and 34% [80,84]. Chlorogenic acids mainly undergo phase II metabolism (in the intestine, liver and/or kidney), being sulphated by sulfotransferases (i.e., SULT1A1 and SULT1A3 isoforms) and glucuronidated by uridine 5′-diphosphate (UDP)-glucuronyltransferases (i.e., UGT1A1 and UGT1A9 isoforms) [79,85]. Furthermore, both primary and secondary metabolites can be conjugated with glycine [76,80]. Several authors found that chlorogenic acids can be excreted by digestive fluids and that they can be recycling by enterohepatic recirculation. Urinary excretion of chlorogenic acids occurs primarily in sulphated, glucuronidated and glycine conjugated form. Apart from that, about 40 other compounds identified as the primary or secondary metabolites of chlorogenic acids are found in urine [76,80,86].

### 2.3. Caffeic Acid

According to Olholf et al. [86] about 95% of caffeic acid is absorbed in the first parts of the alimentary system in humans, i.e., in the stomach and/or small intestine. Most probably, in the stomach caffeic acid is absorbed by passive non-ionic mechanism, whereas in the small intestine, this compound can be absorbed via active transport. Its maximum plasma concentration occurs within 1 h after consumption and decreases quite rapidly [87,88]. After absorption, caffeic acid undergoes enzymatic conjugation, i.e., methylation, sulphation, and glucuronidation by sulfotransferases, UDP-glucotransferases, and catechol-O-methyltransferases, respectively [89]. Manach and colleagues [87] found out that caffeic acid is primarily excreted in urine (up to 27%). Free caffeic acid that has not been absorbed in the small intestine can be reduced (by gut microbiota) into dihydrocaffeic acid (3-(3,4-dihydroxyphenyl)-propionic acid) which in turn is transformed into 3-(3-hydroxyphenyl)-propionic acid and 3-phenylpropionic acid. After that, the latter compounds are absorbed in the colon. In the liver, they undergo beta-oxidation, and in consequence, benzoic acid and hydroxybenzoic acid are produced. Benzoic acid and hydroxybenzoic acid conjugated with glycine and the obtained metabolites (i.e., hippuric acid and 3-hydroxyhippuric acid) are excreted with urine [90].

### 2.4. Trigonelline

In humans, plasma levels of trigonelline vary depending on the coffee type, and the amount of consumed coffee is a reliable predictor of plasma trigonelline values [91,92]. Considerably higher C_max_, C_min_, C_avg_, AUC_0-24_ values as well as the 24-h total excretion concentrations for trigonelline were detected in subjects that drank three cups of espresso coffee per day when compared to volunteers drinking only one cup of espresso coffee with or without two cocoa-based products containing coffee [91]. Most probably, absorption of trigonelline took place primarily in the small intestine, and the circulating levels of this compound are significantly elevated within the first hours after coffee consumption [91,93]. Trigonelline levels seem to drop to the basal values after 24 h post-coffee exposure, though Bresciani et al. [91] suggested a sort of plasma accumulation after its repeated administration. This feature can be related to the long elimination half-life (ca. 5 h) [94]. It seems that trigonelline plasma levels were influenced by food and age since nonfasting subjects presented its higher values (by 20%) as compared to the fasting ones. Trigonelline plasma concentrations augmented with age (i.e., by 9%/10 years) [92]. Furthermore, sex-dependent differences in trigonelline pharmacokinetics were observed, with higher C_max_ or C_avg_ values in women [91,93]. In experiments by Yuyama and colleagues [95,96], about 10% of the oral dose of trigonelline was excreted in urine as *N*′-methyl-2-pyridone-5-carboxylic (an oxidation product), and ca. 20% was recovered unchanged. Sex-dependent differences in relation to trigonelline renal excretion have been detected [93].

### 2.5. Kahweol and Cafestol

There is scarce availability of data on pharmacokinetics of cafestol and kahweol in humans. Most of them are from studies by de Roos et al. [97] carried out on healthy ileostomy volunteers. The authors found that ca. 30% of consumed cafestol is broken down by gastric juices, whereas about 64–70% of ingested cafestol is absorbed, with duodenal absorption ranging between 84 and 93%. Furthermore, it was observed that only 1.2% of ingested cafestol is excreted in a form of glucuronidated or sulphated conjugates in urine. As for kahweol, when consumed, ca. 70–73% of this compound is absorbed by healthy ileostomists, with the small intestine absorption within the range of 91–95%. The rest of it is degraded by gastric enzymes. Only insignificant amount of consumed kahweol (i.e., 0.4%) is excreted in a glucuronidated or sulphated form in urine.

## 3. Neurodegenerative Diseases

Neurodegenerative disorders encompass a heterogeneous group of diseases that are related to progressive deterioration of the structure and functioning of the central or peripheral nervous system. Neurons, synapses, glial cells, and their networks are affected. Usually, accumulation of pathological proteins in both neurons and glial cells of the human brain and the spinal cord or their extracellular depositions (plaques) are responsible for the nervous system damage. Classification of the neurodegenerative disorders depends on clinical symptoms, impaired brain areas, affected cell types, altered proteins, and etiology. Patients suffering from these diseases present movement disorders (such as hyper- or hypokinesia, cerebellar dysfunctions, and problems with the upper and lower motor neurons), cognitive decline, dementia, and disturbances in many high-order brain functions. Affected brain areas have signs of atrophy and/or defective metabolic activity [98].

### 3.1. Dementias, Including Alzheimer’s Disease

According to the literature data [99], about 50 million people worldwide currently suffer from dementia. This number is increasing all the time due to population growth and aging, and most probably by 2050, it will be doubled [100]. Dementia, defined as an acquired chronic or progressive cognitive impairment is one of the major causes of dependence, disability and even mortality in elderly people. In this syndrome, deterioration of cognitive functions is far beyond the aging-related physiological decline, and it affects profoundly the quality of patient’s life. Though the consciousness of people with dementia is not usually disturbed, they present deteriorated learning capacity, reduced visuospatial, language, calculation, and judgment skills as well as worsened memory, thinking, and orientation. Furthermore, their emotional control, social behavior, and motivation are also negatively changed. There are several different forms of dementia, including Alzheimer’s disease (about 60–70% of all cases), vascular dementia, dementia with Lewy bodies, frontotemporal dementia, mixed dementia, and others [101].

Typical, sporadic Alzheimer’s disease with a late onset is usually associated with an interplay between environmental factors and genetics. Apart from that, a familial form of Alzheimer’s disease is also known, which is related to mutations in amyloid precursor protein (APP), PS1 presenilin 1 (PS1), and presenilin 2 (PS2) genes [102]. It has been suggested that cognitive impairment in patients with Alzheimer’s disease is induced by the progressive degeneration of the neocortex [103], basal forebrain [104], and the limbic system [105], with an initial damage of synapses, followed by deterioration of axons, and atrophy of dendrites and somas [106,107,108,109]. Both “positive” and “negative” lesions, with their characteristic distribution, are implicated in the pathogenesis of Alzheimer’s disease. Amongst the positive ones, amyloid plaques and neurofibrillary tangles seem to be most important, but neuropil threads and dystrophic neurites with hyperphosphorylated protein tau are also mentioned. They may co-exist with formation of Hirano bodies, congophilic amyloid angiopathy, astrogliosis, microglial cell activation, and granulovacuolar degeneration. As for the negative lesions, neuronal, synapse, and neuropil loss are observed.

Amyloid plaques are accumulated outside neurons, mainly in the isocortex. However, in advanced cases, they can also be found in the subcortical structure. Amyloid plaques mostly consist of the abnormally folded amyloid beta (Aβ) peptide with 40 or 42 amino acids. They are produced during metabolism of the amyloid precursor protein. Since Aβ peptide with 42 amino acids is less soluble and presents higher rate of fibrillization, it is more abundant within the plaques [110]. Unfortunately, Aβ pathology is not a reliable indicator of the disease progression, since it relatively quickly reaches the plateau level [111]. Neurofibrillary degeneration seems to be a better marker. A number of studies have revealed that the density and distribution of the neurofibrillary tangles correspond to the severity of the disease. The intracellular neurofibrillary tangles consist of paired helical filaments that are built of the aberrantly misfolded and hyperphosphorylated microtubule-associated protein tau. Neurofibrillary pathology begins in the allocortex of the medial temporal lobe, and then, it spreads to the associative isocortex. The primary sensory, motor, and visual areas are involved only at the latest stage of the disease [110]. It has been suggested that Aβ plaques perturb communication between neurons in synapses, and consequently, they contribute to cell death and brain atrophy. Tau tangles most probably inhibit transportation of nutrients and other vital compounds inside neurons. Furthermore, it is believed that both amyloid plaques and neurofibrillary tangles stimulate immune cells in microglia, which results in chronic inflammation. Thus, it is certain that both amyloid and tau pathologies are crucial for the development of Alzheimer’s disease. However, scientists are not unanimous in relation to which of them is the primary process. The tau hypothesis of Alzheimer’s disease assumes that the hyperphosphorylation of tau is the predominant mechanism [112], whereas according to the amyloid hypothesis of Alzheimer’s disease, accumulation of the amyloid plaque as a result of imbalance between production and clearance of Aβ peptide is the primary cause of the disease with development of neurofibrillary tangles, neuronal dysfunction, and degeneration as the secondary processes [113]. In fact, mutations in Aβ genes can be causative factors of Alzheimer’s disease [114], while tau mutations by themselves do not induce this disease [115]. Available literature provides also other explanations for Alzheimer’s disease development, suggesting that progressive loss of cholinergic neurons with subsequent reduction in acetylcholine levels in the cerebral cortex [116,117], dysfunction of the brain mitochondria [118], reduced cerebral blood flow [119], or imbalance in metabolic processes (i.e., diabetes, obesity, hypercholesterolemia) [120,121] contributes at least partially to Alzheimer’s disease onset. Furthermore, patients with Alzheimer’s disease present signs of neuroinflammation [122] and oxidative stress [123].

For the time being, there is no effective prophylactic or causative therapy for Alzheimer’s disease. Symptomatic drugs are used, including cholinesterase inhibitors (i.e., donepezil, rivastigmine, and galantamine) and memantine (i.e., an antagonist of the *N*-methyl-ᴅ-aspartate (NMDA) receptor). Additionally, antipsychotics and antidepressants for the treatment of behavioral symptoms are prescribed [124].

### 3.2. Parkinson’s Disease

Parkinson’s disease is another progressive and degenerative disorder that globally affects more than 6 million people [125]. It is manifested by both motor and nonmotor symptoms. The motor symptoms include resting tremor (usually unilateral in extremity, though the head, jaw, and tongue can also be involved), bradykinesia, postural instability, and rigidity. Spontaneous movement are significantly decreased, with the loss of facial expression, reduced blink rate, and impaired spontaneous swallowing that results in sialorrhea. Furthermore, hand movements are limited and periods of “freezing” and gait changes are noted. Patients with Parkinson’s disease may experience propulsion or retropulsion, and festination [126,127]. Amongst the nonmotor symptoms cognitive decline, anosmia, depression, anxiety, dysautonomia, gastrointestinal and urinary complaints, sleep disturbances, and orthostatic hypotension are listed [128,129,130,131,132]. On the cellular level, substantia nigra and locus coeruleus depigmentation as well as neuronal deficits in the pars compacta of the substantia nigra are observed. These pathologies seem to be related to apoptosis and autophagy [133]. Furthermore, Lewy bodies or Lewy neuritis, i.e., cytoplasmic abnormal aggregations of misfolded α-synuclein, are detected in certain regions of the central and peripheral nervous system [134], including basal and celiac ganglia, locus coeruleus, dorsal motor nucleus of the vagus, olfactory bulb, or the intermediolateral nucleus in the spinal cord [135,136]. It was demonstrated that phosphorylation and fibrillization of α-synuclein induce neuronal death [137]. There is a general notion that the neurodegeneration in Parkinson’s disease concerns mainly dopaminergic neurons and thus, it has a noxious impact on dopamine levels and dopamine-related neurotransmission [138,139]. However, neuronal deficits and Lewy formations have been found in the noradrenergic, serotonergic, and cholinergic systems, as well [140]. Therefore, the abovementioned pathways can also be affected. Though in some patients Parkinson’s disease has a genetic origin, the primary cause of the most Parkinson’s disease cases has not been discovered yet. Inflammation, oxidative stress, mitochondrial dysfunction along with disturbances in protein handling and in activity of calcium channels are mentioned as the contributing factors to the observed neuronal loss [134].

Currently, there is no effective cure for Parkinson’s disease. Prescribed medications help to alleviate symptoms and improve the quality of patient’s life. Most of them stimulate dopaminergic neurotransmission. Levodopa, i.e., a precursor of dopamine, is still considered as the most potent active substance that controls Parkinson’s disease manifestations. Usually, it is given with carbidopa that increases its bioavailability and inhibits its peripheral metabolism. Dopaminergic agonists (pramipexole, ropinirole, rotigotine, or apomorphine) activating dopaminergic receptors as well as inhibitors of catechol-O-methyl transferase (entacapone, opicapone) and monoamine oxidase aldehyde dehydrogenase B (rasagiline, selegiline, safinamide) that slow down enzymatic degradation of levodopa and dopamine are also used. Rigidity, dystonia, and tremor are usually treated with anticholinergic drugs (trihexyphenidyl and benztropine), whereas hallucinations and delusions are controlled with antipsychotics, such as quetiapine, clozapine, or pimavanserin [141,142].

### 3.3. Ischemic Stroke

It has been estimated that globally about 13–15 million people undergo stroke each year, which results in more than 5 million deaths [143]. About 85% of strokes are ischemic ones. Ischemic stroke occurs when the blood flow to the brain is decreased. It may be caused by a thrombotic event or an embolic event. In the thrombotic event, the blood flow is obstructed due to vessel problems (i.e., as a consequence of arterial dissection, atherosclerotic disease, fibromuscular dysplasia), whereas in the embolic event, the blood flow is obstructed due to a clot that originated in another location within the body (frequently in the heart) and was dislodged to the brain vasculature. Depending on the affected artery, several ischemic stroke syndromes are diagnosed, including middle cerebral artery infarction, anterior cerebral artery infarction, vertebrobasilar infarction, cerebellar infarction, and lacunar infarction. Thus, the clinical presentation of a given ischemic stroke is different depending on the brain regions that are supplied by the involved vessel. The observed deficits in motor functions and cognition are caused by the loss (necrosis) of brain tissue in the influenced areas. Most frequently, weakness of the face, tongue, and/or laryngeal muscles, speech disorders, contralateral hemiparesis, visual disturbances, impaired coordination and balance, severe headaches, or impaired consciousness are reported [144,145,146,147,148]. The main treatment goal in an acute ischemic stroke is to avoid necrosis of the tissue in the affected region. Therefore, when possible, a thrombolytic compound (i.e., tissue plasminogen activator) is administered. Apart from that, mechanical thrombectomy, aspirin or heparin, and antihypertensive drugs (i.e., labetalol, nicardipine, clevidipine, hydralazine, enalaprilat) are used. In order to obtain neuroprotective effect, drugs should be given as soon as possible after the stroke onset [149,150,151].

There are several mechanisms responsible for the brain sensitivity to ischemia. One of them is the excitatory activity of glutamate. It has been found that ischemia causes a significant decrease in adenosine-5′-triphosphate (ATP), which in consequence disturbs activity of glutamate transporters responsible for removal of glutamate from the synaptic cleft. Elevated level of glutamate leads to overstimulation of glutamate receptors and excessive increase of calcium levels. These processes generate excitotoxicity, neurons damage and their death [152]. Furthermore, acidification of brain tissue observed after stroke worsens the brain injury [153,154]. Most probably, acidosis-mediated stimulation of the so-called acid-sensing ion channels and the subsequent influx of calcium ions are implicated in this pathological mechanism [155]. After ischemic stroke, neuroinflammation, oxidative stress, and disruption of the blood–brain barrier are also detected. Microglia and astrocytes are activated which intensifies production of chemokines and cytokines along with infiltration of leukocytes [156]. Eventually, epigenetic remodeling including DNA methylation and histone modifications may be responsible for memory deficits diagnosed in patients that underwent ischemic stroke [157]. Unfortunately, necrosis of tissues at the site of infarction may instigate further damage of the brain, spreading to the regions anatomically related to that site. This process is called the secondary neurodegeneration [158]. Surprisingly, areas affected by the secondary neurodegeneration share common features with typical neurodegenerative disorders, such as neuroinflammation, progressive neuronal loss, or accumulation of Aβ which is specific to Alzheimer’s disease [159]. It seems that the thalamus is particularly vulnerable to the secondary degeneration after stroke. Its disturbances are detected within few weeks after infarction and can persist for several years. Stroke-induced degenerations in thalamus include neuronal loss, severe glial dysfunction [160,161,162], and Aβ accumulation [163]. Preclinical studies by Ong et al. [164] confirmed that stroke-induced accumulation of Aβ in the thalamus may be connected not only with an increase of the high molecular weight soluble amyloids but also with Aβ oligomers and that this form of Aβ may also be implicated in neuronal loss after stroke. Interestingly, chronic stress [164] or administration of the human bone marrow-derived mesenchymal stem cells [165] in a rodent stroke model aggravate accumulation of Aβ in the thalamus, whereas administration of a γ-secretase inhibitor [166], calcium channel blocker [167], or autophagy inhibitor [168] reduces amounts of Aβ in the thalamus as well as improves functioning of neurons after stroke.

### 3.4. Epilepsy

One of the most common neurological diseases is epilepsy, which affects about 50 million people worldwide. It has been estimated that ca. 5 million people are diagnosed with epilepsy per year [169]. The disease is characterized by recurrent seizures that can be generalized (tonic-clonic, involving both hemispheres and multiple structures) or focal (limited to one hemisphere). Though up to 70% of epileptic patients can be seizure-free taking antiepileptic drugs, there is still a great number of people that do not respond to the available treatment. Drugs are selected individually (usually starting with monotherapy) with several different factors taken into consideration, including seizure type, comorbidities, concomitant drugs, patient’s lifestyle, and their preferences [170].

Hippocampal sclerosis, i.e., pyramidal cell loss in Ammon’s horn, gliosis, granule cell dispersion, and axonal fiber sprouting, has been found in epileptic patients [171,172,173]. Briellmann et al. [174] and Jackson et al. [175] reported a significant reduction in hippocampal volume and altered hippocampal architecture associated with seizure episodes. Most probably, the seizure-induced neuronal death is caused by upregulated glutamatergic neurotransmission (excitotoxicity) which results in extensive influx of calcium ions into cells, osmolytic stress, and stimulation of cell death pathways [176]. Proliferation and hypertrophy of microglia, astrocytes, and oligodendrocytes detected in patients with epilepsy is associated with elevated levels of proinflammatory cytokines in the brain [177,178]. Impairments in the blood–brain barrier as well as changes in the brain vascular system are also observed in epilepsy. However, it has not been determined whether microvessel proliferation and disruption in the blood–brain barrier are the causative factors of seizures or they occur as a consequence of seizures [179,180].

## 4. Neuroprotective Effects of Coffee Bioactive Compounds

Epidemiological studies suggest that regular coffee consumption may be associated with a reduced risk of numerous neurodegenerative disorders (including Parkinson’s disease, Alzheimer’s disease, and neurocognitive decline), though conflicting results have also been reported [181,182,183]. When consumed in moderate amount, coffee may reduce dementia and improve cognitive performance [183,184]. Moreover, habitual coffee consumption can potentially decrease the risk of stroke incidence and stroke mortality [182,183,185] and has positive impact on the course of autoimmune diseases such as multiple sclerosis [183,184]. Caffeine is the most widely investigated coffee component, and benefits from regular coffee intake are typically attributed to caffeine. However, coffee is a mixture of many bioactive compounds and some of them have the potential to produce neuroprotective effects as well. Here, we provide a comprehensive overview of the data from in vitro and in vivo studies on the neuroprotective potential of the main bioactive coffee components. Studies in humans, although limited, are also discussed.

### 4.1. Neuroprotective Effects of Caffeine

Caffeine (1,3,7-trimethylxanthine), because of its chemical structure, is classified as a purine alkaloid and is the dominant physiologically active compound in coffee beans and soft beverages. This methylxanthine belongs to the most favorable used psychostimulant worldwide [7,12,186]. By consuming a cup of brewed coffee (about 430–440 mL), an average of 188 mg caffeine is delivered to the body (range 147–259 mg depending on the genus of coffee beans) [187]. Moderate caffeine intake (3–5 cups/24 h) is associated with reducing fatigue, revised cognitive, and improved alertness, leading to better yield in psychomotor tasks needing quick response [188,189]. Furthermore, studies have shown that caffeine has antioxidant [20,190,191], anti-inflammatory [15,191], anti-cancer [22,24,25], as well as neuroprotective properties. The mechanisms underlying these caffeine activities have been thoroughly investigated over the last decade. In this paragraph, an overview of the most important preclinical and clinical studies that investigated the neuroprotective effects of caffeine has been presented.

*Preclinical studies*. Both neuroprotective effects of caffeine and the mechanism of this action have been examined in different experimental models of central nervous system (CNS) diseases. Preliminary studies on a long-term caffeine administration on behavior of naïve rodents revealed no effect on spatial learning and memory responses [192]. However, later, the protective impact of the chronic caffeine administration on the onset of cognitive impairment in Alzheimer’s mice has been revealed in several works. Costa et al. [193] demonstrated that a 12-month treatment with caffeine averts memory impairment in aging rodents. The caffeine-treated aging mice presented a similar recognition memory as adult mice and an improved recognition memory when compared to their age-matched control animals. Furthermore, it was noted that caffeine prevents the age-depending enhancement in the hippocampal immunocontent of the brain-derived neurotrophic factor (BDNF) and tirosine kinase receptor (TrkB), which might be a mechanism for caffeine’s neuroprotective action [193]. Citied outcomes are corroborated with results of preclinical studies conducted by Arendash et al. [194,195]. This research team demonstrated that giving caffeine in the daily diet to Swedish mutation transgenic mice (animals carrying the mutant APPK_670N,M671L_ gene, APPsw), starting in young adulthood, results in cognitive protection in various tests across a multiple of cognitive domains, such as spatial learning, memory, identification, strategy switching, and working memory. Moreover, these comprehensive cognitive profits did not contribute to the occurrence of undesirable effects, such as disturbances in sensorimotor functions or an increase in the level of anxiety, which may be caused by a single caffeine administration [194,195]. More recent research by Arendesh and co-workers [195] indicated that a long-term moderate caffeine consumption has also a desirable effect on already existing Alzheimer’s disease symptoms in older (18–19 month old) APPsw mice [195]. They observed that aged APPsw rodents after 4–5 weeks caffeine administration in drinking water characterized significantly better working memory in comparison to the control APPsw animal group [195]. In both studies, they indicated, that prolonged caffeine intake decreases hippocampal Aβ levels, which are most likely associated with reduced expression of both PS1 and β-secretase-1, and hence diminished production of Aβ in caffeine-treated APPsw mice [194,195]. Besides, an evidence that observed β-secretase-1 suppression after caffeine treatment involves the cRaf-1/NFκB (nuclear factor κ-light-chain-enhancer of activated B cells) inflammatory pathway was presented [195]. Additionally, the ability of caffeine to impair Aβ synthesis (in a concentration-dependent manner) [194] and to decrease total glycogen synthase kinase 3 (GSK-3) levels (in a concentration- and time-dependent manner) [195] were revealed in the nerve cell cultures SweAPP N2a. As emphasized by the authors, it is also probable that the mechanism of caffeine’s protective effect on cognition may be due to the restoration of adenosine levels to normal in transgenic mice, despite the lack of effect on the density of A_1_ and A_2A_ adenosine receptors [194]. Moreover, they showed that chronic caffeine consumption from adulthood to old age does not provide cognitive benefits in normal mice [195]. These findings are in agreement with the outcomes of Dall’Igna et al. [196,197] showing that chronic as well as sub-chronic caffeine administration resulted in a robust protection against Aβ peptide toxicity in cerebellar neuron cultures [196] and prevented the Aβ-induced cognitive impairment [197]. Recent in vitro analyses conducted by Giunta et al. [198] also showed that caffeine prevents neuroblastoma cell death induced by co-exposure to Aβ and aluminum chloride (AlCl_3_). Additionally, they demonstrated, that caffeine treatment, through a non-selective blockade of A_1_ and A_2A_ adenosine receptors, inhibits the co-neurotoxicity of Aβ and AlCl_3_ [198].

The impact of prolonged caffeine administration on memory impairment and oxidative stress generated by aging in rats was investigated by Leite et al. [199]. The obtained outcomes indicated that the memory deficits appearing with age are reversed by oral administration of caffeine. In addition, biochemical studies demonstrated that the applied treatment contributes to the normalization of the enhanced levels of oxygen and nitrogen reactive species (ROS and RNS, respectively) and the inhibited Na^+^/K^+^-ATPase activity noted in the brain of elderly rats [199]. Antioxidant-like properties of chronic caffeine administration as a mechanism of its protective effect on memory deficits, neuroinflammation and neurodegeneration induced by d-galactose treatment were indicated by Ullah et al. [191]. Results of these studies demonstrated that prolonged caffeine administration in the d-galactose-treated rats: (1) reverses oxidative stress via decrease of 8-oxoguanine; (2) attenuates phoshorylation of key stress-responsive kinases level, i.e., C-Jun *N*-terminal kinases (p-JNK); (3) normalizes the level of inflammatory mediators, such as cyclooxygenase-2 (COX-2), nitric oxide synthase-2 (NOS-2), tumor necrosis factor α (TNF-α), and interleukin-1β (IL-1β); (4) prevents apoptosis and neurodegeneration (decreased level of cytochrome C, Bax/Bcl2 ratio, caspase-9, caspase-3, and PARP-1); (5) improves the pre-synaptic proteins synaptophysin and post-synaptic density proteins (PSD95) level, and (6) improves spontaneous alternation behavior [191]. Beneficial caffeine effects on the parameters of oxidative stress have also been demonstrated in in vitro examinations using human neuroblastoma cells exposed to the toxic effect of Aβ and AlCl_3_. In addition, Giunta et al. [198] presented caffeine ability to prevent the activation of the NF-κB pathway, elevation of both β-secretase-1 and APP levels, and ability to inhibit ROS production. Caffeine effects in the cell toxicity model were similar to these noted for an antioxidant–*N*-acetylcysteine and a metal chelator–desferrioxamine [198].

In 2014, Laurent et al. [200] provided the evidence that chronic caffeine intake in drinking water is sufficient to prevent the development of spatial memory deficits in a mice model of progressive Alzheimer’s disease-like tau pathology. Further, the improvement of memory was connected with decreased phosphorylation of hippocampal tau and proteolytic fragments. In addition, in the hippocampus of THY-Tau22 mice, caffeine reduced levels of several pro-inflammatory and oxidative stress markers (i.e., CD45, TLR2, CCl4, and TNF-α) which were upregulated in animals with Alzheimer’s disease [200]. The evidence for the protective activity of caffeine against oxidative stress and Alzheimer’s disease-like pathology has also been presented by Prasanthi et al. [201]. They demonstrated that caffeine treatment reversed changes induced by cholesterol-enriched diet, i.e., it decreased ROS generation, glutathione depletion, as well as Aβ synthesis, whereas it increased adenosine A_1_ receptors concentration in the rabbit hippocampus [201]. Another hypothesis assumes that increased cerebrospinal fluid (CSF) production is a possible mechanism underlying caffeine’s protective effect against Alzheimer’s disease. Han et al. [202] showed that the long-term caffeine consumption might induce ventriculomegaly and intensify production of CSF as a result of the enhancement of expression of Na^+^/K^+^-ATPase and cerebral blood flow (CBF). In contrast, acute caffeine administration has an opposite effect on the production of CSF [202] (for review see [203]).

Numerous studies have attempted to determine the effects of caffeine consumption on the development and course of Parkinson’s disease in various animal models. It has been demonstrated that caffeine attenuated dopaminergic lesions caused by 1-methyl-4-phenyl-1,2,3,6-tetrahydropyridine (MPTP) [204,205,206,207,208,209], 6-hydroxydopamine (6-OHDA) [210,211], and pesticides (paraquat/maneb) [212]. What is more, caffeine pre-treatment decreased neuronal damage and improved motor activity [204] and attenuated dopamine loss [208] and microglia activation in the substantia nigra [213]. Additionally, Sonsalla et al. [213] recorded that both caffeine administration for 1 week and 3 weeks after initiating MPTP infusion (the early stage of loss of nigrostriatal dopamine and the late stage of loss of nigrostriatal dopamine, respectively) decreased the decline of nigral cells in rats by 94% and 69%, respectively. Reduction in the loss of nigrostriatal dopamine neurons in rats was also observed when caffeine was taken orally after MPTP administration [213]. In turn, an acute caffeine pretreatment was demonstrated to be only partially beneficial against neurotoxic changes obtained in the MPTP [209] and 6-OHDA [214] of Parkinson’s disease rodent models. Some of these studies have shown that the observed protective activity on dopaminergic neurons/dopamine levels is dose-dependent, and that the maximum neuroprotective effect is achieved after caffeine administration at a daily dose of 10 mg/kg. Moreover, this effect was grater in young (10 weeks) mice in comparison to the old (6–9 months) ones [208]. Moreover, Xu et al. [209] indicated that caffeine metabolites (both theophylline and paraxanthine) also significantly attenuated the MPTP-induced dopamine depletion in mice, thus also providing neuroprotective effects in this model of Parkinson’s disease.

The exact mechanism by which caffeine provides neuroprotection against toxins is still unclear. The most prominent theory about antiparkinsonian potential of caffeine is that this methylxanthine acts as an antagonist of adenosine A_2A_ receptors. In the substantia nigra, caffeine via competitive inhibition of these receptors might prevent the adenosine-mediated neuroinflammatory actions [213]. Laboratory data showed that various A_2A_ antagonists (both non-selective and selective) protect against acute toxin exposure in Parkinson’s disease models [208,212,215,216,217,218,219]. Through blockade of adenosine A_2A_ receptors, caffeine inhibits activation of adenylyl cyclase and consequently protein kinase A. Therefore, it restrains the extracellular calcium influx into a cell and reduces the excitotoxic glutamate release in the CNS [204,218,220,221]. Moreover, Morelli et al. [222] indicated that caffeine by blocking A_2A_ receptors and reducing glutamate release contributes to attenuation of microglia activation and production of both cytokines and free radicals, hence precluding further damage of striatal and nigral neurons [222]. Caffeine is also capable to bind to adenosine A_2A_ receptors situated on astroglial cells, thereby inhibiting their activity and regulating the neuroinflammation generated by astroglia in the vicinity of dopaminergic neurons [214,217]. The essential role of neuronal adenosine A_2A_ receptors in chronic neurodegeneration was confirmed in mice with the A_2A_ receptors knockout. Such animals showed resistance to dopaminergic neuron damage caused by a chronic [223], although not an acute [219] exposure to MPTP. However, adenosine A_1_ receptors antagonism did not produce the neuroprotective effect observed after caffeine treatment [215]. Likewise, neurochemical and immunohistochemical studies conducted in recent years indicated that long-term caffeine intake in various animal models of Parkinson’s disease: (1) increased dopamine levels, (2) reversed the enhanced dopamine and noradrenalin levels in striatum, (3) improved the hippocampal neuronal viability, (4) increased tyrosine hydroxylase immunoreactivity in the striatum, (5) reduced the number of immunopositive cells for histone deacetylase, (6) decreased the level of pro-inflammatory cytokines, such as TNF-α and IL-1β [204,210,212,213,214,215].

The anti-ischemic effect of caffeine has been examined using animal models of ischemic brain injury. Rudolphi et al. [224] observed that chronic oral pretreatment with caffeine greatly reduces the degree of ischemic necrosis of pyramidal cells of the CA1 hippocampal area in Mongolian gerbils subjected the bilateral carotid occlusion. Moreover, this study outcome provided the evidence that a caffeine-induced upregulation of A_1_ adenosine receptors in the CNS impairs the level of experimentally induced ischemic brain injury [224]. Similar outcomes following chronic treatment of mice with very low doses of caffeine were reported by Georgiev et al. [225]. In the study by Evans et al. [226], caffeine administered to the cortex 60 min prior to the development of ischemia decreased the ischemia-induced attenuation of the amplitude of recorded somatosensory evoked potentials and accelerated recovery to control levels [226].

The effect of caffeine on ischemic neuronal injury in rats using magnetic resonance imaging (MRI) and histopathological examination was investigated by Sutherland et al. [227]. Acute caffeine-treated animals exhibited accelerated changes in the MRI scans, while quantification of the histopathological evidence revealed no meaningful distinction in neuronal injury in any brain region in comparison with control-ischemic rats. Moreover, chronic caffeine-treated rodents had significantly minor neuronal damage in all sensitive brain areas (including cerebral cortex, striatum, and hippocampus) than either of the other ischemic rats’ groups. Additionally, on the basis of the obtained results, they indicated that protection against ischemic injury after chronic administration of caffeine might be effectuated via an enhancement in the concentration of adenosine receptors [227] in the CNS, which is consistent with the caffeine neuroprotection mechanism in ischemic brain injury proposed by Rudolphi et al. [224].

Therapeutic activity of caffeine treatment in neonatal hypoxic-ischemic (HI) injury model was studied by Alexander et al. [228]. Results of this research showed that caffeine-untreated HI animals had significant deficits in the Morris water maze test, which have been attenuated by caffeine administration immediately after the induction of HI. Furthermore, they also found a decrease in cortical volume in the HI saline-treated animals, while cortical volume in the HI caffeine-treated animals was intermediate. Similarly, Kilicdag and co-workers [229] observed the reduced neuronal apoptosis in the developing brain in caffeine-treated rats in a HI neonatal model. Moreover, later findings presented by Potter et al. [230] supported the continued investigation of caffeine as a neuroprotectant in a preterm model of HI. All of these research teams concluded that caffeine might be efficacious in extenuating ischemic brain injury [228,229,230].

Summary of in vivo studies on the neuroprotective effects of caffeine is presented in Table 3. *Clinical studies*. A case-control study carried out by Maia and de Mendonça [231] with 74 patients with Alzheimer’s disease and 72 healthy subjects aimed to answer the question whether caffeine intake protects from Alzheimer’s disease [231]. Consequently, the authors calculated the average daily caffeine intake (mg/day) by estimated caffeine content in various food products, which are widely recognized as the primary sources of this methylxanthine (e.g., instantaneous coffee–60 mg, decaffeinated coffee–3 mg, espresso coffee–100 mg, instantaneous tea–20 mg, leaf tea–30 mg, and cola-drinks–18 mg) and counted how many dosages each patient consumed for the period of 20 years before diagnosis of Alzheimer’s disease and the period from early adulthood to 20 years before diagnosis of Alzheimer’s disease, as well as for the period after the diagnosis of Alzheimer’s disease until the time the questionnaire. This study showed that caffeine intake was inversely correlated with the hazard ratio of developing Alzheimer’s disease–an increased caffeine consumption was associated with a 60% reduction in the risk of Alzheimer’s disease (average consumption was 199 ± 136 mg/day in healthy subjects compared to 74 ± 98 mg/kg in patients with Alzheimer’s disease) [231]. Caffeine’s beneficial effects in Alzheimer’s disease patients were also observed in the Canadian Study of Health and Aging. A prospective analysis of risk factors for Alzheimer’s disease was conducted on a group of 1023 individuals aged 65 years or older in 1991–1992, and its outcomes showed that coffee consumption was associated with a reduced risk of Alzheimer’s disease and amounted to 31% [232]. Interesting results were also obtained by Eskelinen and co-workers [233,234] in studies assessing the association between the long-term coffee consumption at midlife and Alzheimer’s disease/dementia risk in late-life. After an average follow-up of 21 years, in the group of 1409 individuals (534 men and 875 women) aged 50 years in 1972–1977, moderate coffee drinkers (3–5 cups/24 h) had lower risk of Alzheimer’s disease and dementia (by 62–64% and 65–70%, respectively) in comparison with low coffee consumers (0–2 cups/24 h). Results from this clinical study indicate that regular consumption of coffee/caffeine seems to be protective for Alzheimer’s disease and dementia [233,234]. Likewise, several meta-analyses [235,236] and some systematic reviews [237,238,239] demonstrated an inverse association between cognitive impairment/decline and the risk of Alzheimer’s disease. Furthermore, there are several trials in which caffeine seemed to have no beneficial properties in patients with Alzheimer’s disease/dementia. In a large prospective population study (4197 women and 2820 men aged 65 years and over) by Ritchie et al. [240] no impact on dementia incidence in women and men and no association between caffeine intake and cognitive decline in men were found. In turn, in women with a high level of caffeine intake (>3 cups/day) a lesser decline in the visuospatial memory over 4 years than in women consuming ≤1 cup/day was noted. Moreover, it was noticed that the protective activity of caffeine increased with age [240]. The meta-analysis of the observational epidemiological research by Kim et al. [241] also showed no significant relationship between caffeine intake from coffee and the hazard ratio of cognitive disorders, including Alzheimer’s disease and dementia, as well as cognitive decline, in spite of the 18% tendency to reduce the risk of developing these disorders.

Numerous clinical studies and meta-analysis/systematic reviews have also linked caffeine use with a lower risk of Parkinson’s disease. The possible association between Parkinson’s disease risk and caffeinated beverages has been examined since the early 1970s. A significant negative relationship was found for caffeine consumption and hazard ratio of Parkinson’s disease in one of the recent systematic review and meta-analysis–in caffeine drinkers the relative risk of Parkinson’s disease was reduced by approximately 30–38% [242,243,244,245]. Moreover, in 2014 Qi and Li [245] presented the dose-response meta-analysis which suggested a linear association between the decreased risk of Parkinson’s disease and caffeine use, and a non-linear relationship between the decreased risk of Parkinson’s disease and coffee consumption. A five-time lower risk of developing Parkinson’s disease in 45–68 year old people drinking coffee in the amount of ≥794 g/day (which corresponds to 421 mg of caffeine per day) and a lower risk of Parkinson’s disease depending on the amount of consumed caffeine, was reported by Ross et al. [246] based on 27 years of follow-up American Japanese. Convergent results were obtained by Hu et al. [247] in a nearly 13-year control study involving about 14,500 people (approximately 62 years old). The Parkinson’s disease hazard ratio was estimated at 1.00, 0.55, and 0.41 for subjects drinking 0, 1–4 and ≥5 cups of coffee per day, respectively [247]. Liu et al. [248] noted that the level of Parkinson’s disease risk reduction is similar in 61 year old women and men consuming ≥5 cups of coffee a day for 10 years. Similarly, Hu et al. [247] reported that the inverse relationship between coffee consumption and the Parkinson’s disease hazard ratio did not differ significantly between men and women in Finland. Palacios et al. [249] indicated that men who consumed ≥2 cups of coffee/day (i.e., 274 mg/day of caffeine) had a lower risk of Parkinson’s disease than women who consumed 3.2 cups of coffee/day (i.e., 435 mg/day of caffeine) (50% and 40% lower risk of Parkinson’s disease, respectively).

In 2011, Altman et al. [250] demonstrated that caffeine may have positive effects on some motor as well as nonmotor aspects in patients suffering from Parkinson’s disease. Moreover, the maximum tolerated dose of caffeine in Parkinson’s disease subjects was 200–400 mg/day [250]. A year later, Postuma et al. [251] in a randomized, controlled trial showed that administration of caffeine at a dose of 200 mg/day for 3 weeks followed by a further 3 weeks at a dose of 400 mg/day significantly improved the overall unified Parkinson’s disease rating scale and motor manifestation (by 4.7 and 3.2 points, respectively). However, results of these studies are in contrast to the recent randomized trial that indicated that caffeine did not produce sustained motor improvement in Parkinson’s disease [252].

Based on the cited clinical studies, meta-analyses and systematic reviews, it is not possible to establish the biological mechanism(s) behind the correlation between coffee/caffeine intake and the risk of Alzheimer’s disease/dementia and/or Parkinson’s disease. Tan et al. [253] analyzing the association between caffeine consumption and hazard ratio of Parkinson’s disease in both fast and slow caffeine metabolizers suggested that both caffeine and its major metabolite, paraxanthine, have neuroprotective properties. These observations supported experimental evidence obtained in animal models (see preclinical studies). Furthermore, several studies showed that decaffeinated coffee consumption was not associated with neurodegenerative disorders risk, including Alzheimer’s disease and Parkinson’s disease [29,249,254]. Therefore, it can be assumed that caffeine is responsible for the observed inverse correlation between coffee intake and the hazard ratio of Alzheimer’s disease and Parkinson’s disease incidents.

Until recently, coffee was classified as one of the cardiovascular risk factors [255,256,257,258,259]. While, caffeine is known to increase peripheral vascular resistance, but also to reduce blood flow in the brain through its vasoconstrictive effects and consequently poses a risk of hypertension (one of the risk factors of stroke) [260], some epidemiological and cohort studies, as well as meta-analysis found there was no significant association between coffee consumption and stroke risk [261,262,263,264,265,266], and several showed a prophylactic effect of coffee consumption on stroke incidence [18,185,267,268,269]. In turn, a study conducted by Mostofsky et al. [270] found an increase in the hazard ratio of an ischemic stroke within 60 min after drinking coffee. Likewise, an acute increase in the risk of ischemic stroke was observed immediately after drinking coffee by Washio et al. [271], but as these authors emphasized, the reason of observed coffee impact may be caused by other factors rather than an elevation in pressure in the cerebral circulation [271].

In 2011, Larsson and Orsini [272] published results of meta-analysis involving 11 prospective studies (a total of 479,689 individuals and 10,003 stroke incidents) which showed a non-linear connection between coffee consumption and the hazard ratio of stroke. In comparison to the absolute risk of total stroke, the relative risk of total stroke amounted to 0.87, 0.84, 0.88, and 0.94 for 2, 3–4, 6, and 8 cups of coffee per day, respectively. Additionally, estimated hazard ratios were suchlike for hemorrhagic and ischemic stroke [272]. A non-linear relationship between coffee consumption and a lower risk of stroke (relative risk 0.80, 95% confidence interval 0.75 to 0.86) was also presented by Poole et al. [18] in umbrella review of meta-analyses (including 201 meta-analyses of observational studies, 67 unique health outcomes, and 17 meta-analyses of interventional studies). A 5% and 15% reduction in a relative hazard ratio of stroke with an average consumption of 5 and 3.5 cups per day versus non-drinkers, respectively, were noted by Ding et al. [267] in a large meta-analysis of 36 cohort studies (36,352 patients with cardiovascular diseases including stroke). Likewise, a prospective study by Larsson [255] confirmed an inverse relationship, but not very marked, between moderate coffee drinking and the risk of stroke.

Otherwise, in some large cohort studies/meta-analyses the association between coffee consumption and the risk of stroke in women and in men was assessed. Larsson and Orsini [272] found that hazard ratios were similar for women and men at lower coffee intake (≤2 cups per day). These results are consistent with those obtained by Lopez-Garcia et al. [261] in the cohort study of women, in which they indicated that long-term coffee drinking was not associated with an increased risk of stroke in women. Furthermore, coffee intake may modestly decrease hazard ratio of stroke in that sex. In this research, women who drank moderate to high amounts of coffee had a lower risk of stroke than women who consumed <1 cup/month coffee (relative risks of stroke: 0.98, 0.88, 0.81, and 0.80 for women drinking 1–16 cup/month, 20–28 cups/month, 60–90 cups/month and ≥120 cups/month, respectively) [261]. As for men, when coffee drinkers were compared to non-coffee drinkers, the stroke risk ratio for those drinking 1–6 cups per week, 1–2 cups per day, and ≥3 cups per day were estimated at 0.78, 0.67, and 0.45, respectively [264]. To explain the likely causal association and elucidate the mechanisms underlying caffeine’s protective effects on stroke, further studies are required.

Both clinical and preclinical studies have shown a beneficial effect of the combination of caffeine and alcohol (caffeinol) in acute ischemic stroke. Strong et al. [273] indicated that co-administration of a low dose of ethanol and caffeine protects the CNS from damage produced by focal ischemia in rats. Moreover, caffeine at a dose of 6 mg/kg with ethanol at a dose of 0.2 g/kg in the caffeinol were effective in decreasing volume of cortical infarct and behavioral dysfunction after reversible common carotid/middle cerebral artery occlusion in rat [274]. Beneficial therapeutic effects as well as safety and tolerability of caffeinol observed in animal studies were later examined and confirmed in clinical research [275,276]. Zhao et al. [277] based on in vivo studies results suggested that observed anti-excitotoxic activity may be the possible anti-ischemic effect of caffeinol, and caffeine can augment anti-ischemic properties of the NMDA receptors antagonists [277].

Although caffeine is a widely used psychoactive substance around the world, its potential therapeutic value has only recently been seriously explored in Alzheimer’s disease, dementia, Parkinson’s disease as well as other cognitive impairments. Animal and human studies showed significantly positive effects of caffeine intake with dose-dependent improvement.

### 4.2. Neuroprotective Effects of Chlorogenic Acid

Chlorogenic acid is a polyphenol that can be found in fruit, vegetables, spices, olive oil, wine, tea, and especially in coffee. Both caffeinated and decaffeinated coffee contains a large amount of chlorogenic acid (70–350 mg per cup of coffee), which makes it one of the most abundant polyphenols in a diet of coffee-consuming populations [278]. Due to a wide distribution in the human diet, chlorogenic acid has gained much research attention. Numerous studies have shown that it exerts multiple health-beneficial effects such as anti-inflammatory, hepatoprotective, cardioprotective, chemopreventive, antidiabetic, and anti-obesity activities. There is also mounting evidence that chlorogenic acid has neuroprotective properties and it appears that its regular intake may reduce risk of neurodegenerative diseases and improve cognition [73,278,279].

*Preclinical studies*. The neuroprotective effects of chlorogenic acid are linked mainly with its ability to reduce oxidative stress. Like other polyphenols, it has free radical scavenging activity and metal-chelating properties [280], and there is considerable in vitro evidence demonstrating protective effects of chlorogenic acid against neuronal damage caused by oxidative stress. For instance, Cho et al. [281] showed that chlorogenic acid suppressed the H_2_O_2_-induced PC12 cell death. The protective effect was related to the attenuation of intracellular ROS accumulation and the inhibition of JNK and p38 MAPK activation. In the study by Kim et al. [282], chlorogenic acid reduced apoptosis in primary cortical neurons by inhibiting the H_2_O_2_-induced downregulation of anti-apoptotic proteins Bcl-2 and Bcl-XL as well as by blocking the H_2_O_2_-induced pro-apoptotic cleavage of caspase-3 and pro-poly(ADP-ribose) polymerase (pro-PARP). In addition, it increased the expression of the antioxidant enzyme–NAD(P)H quinone oxidoreductase (NQO-1). In this study, it was also demonstrated that the neuroprotective effects of caffeinated and decaffeinated coffee were similar, which suggests that other compounds than caffeine (e.g., chlorogenic acids) may be responsible for the neuroprotective properties of coffee [282]. Similar results were obtained by Chu et al. [283] who reported that green and roasted coffees (regular and decaffeinated) protected primary neuronal cells against the H_2_O_2_-induced oxidative damage and improved their survival by inhibiting the extracellular signal-regulated kinase-1 and -2 (ERK1/2) activation. Of note, there was a significant correlation between chlorogenic acid content and the neuroprotective efficacy of the tested samples [283]. In other studies, chlorogenic acid attenuated the H_2_O_2_-induced neurotoxicity, scavenged hydroxyl radical, decreased ROS production in neuro-2A cells [284], and attenuated the H_2_O_2_-induced increases in malondialdehyde (MDA) and ROS levels in rat brain slices [285]. A protective effect against the H_2_O_2_-mediated oxidative insult was also reported in rat pheochromocytoma cells. In this study, chlorogenic acid provided neuroprotection via directly neutralizing free radicals and indirectly inducing the endogenous antioxidant enzymes by activation of nuclear factor erythroid 2–related factor 2 (Nrf2) [286]. Similarly, chlorogenic acid protected against the aluminum-induced cytotoxicity in primary hippocampal neuronal cells by decreasing ROS production and by increasing the expression of Nrf2 and its target phase 2 enzymes [287].

The antioxidant properties of chlorogenic acid also contributed to its neuroprotective effects against the L-buthionine-(S,R)-sulfoximine-induced damage in cultured retinal ganglion cells [288], methylmercury-induced apoptosis in PC12 cells [289], and FeSO_4_-evoked oxidative stress in rat whole brain homogenates [290]. Furthermore, chlorogenic acid protected cultured cerebellar granule neurons from death induced by sodium nitroprusside (SNP)—a NO donor [291] and reduced the SNP-induced increase in MDA content in rat brain homogenates [290]. It also decreased NO level in cerebral neurons exposed to SNP, suggesting that its protective effects against the NO-induced neurotoxicity is likely due to direct free radical scavenging activity [291].

It is widely known that chronic neuroinflammation is closely associated with the pathogenesis of neurodegenerative diseases. Chlorogenic acid was found to reduce neuroinflammation and neurotoxicity in SH-SY5Y cells caused by toxic factors released from activated microglia and astrocytes. Moreover, it decreased production of pro-inflammatory cytokines (TNFα and IL-6) from lipopolysaccharide (LPS)/interferon-γ-stimulated microglia and THP-1 cells, as well as from interferon γ-stimulated astrocytes and U373 cells [292].

Chlorogenic acid was also reported to protect neurons from excitotoxic insults. These are important observations as the glutamate-mediated neurotoxicity is considered to play a crucial role in several neurodegenerative conditions, especially in Alzheimer’s disease, Parkinson’s disease, ischemic stroke, and epilepsy [293]. Oboh et al. [290] showed that it significantly reduced lipid peroxidation in quinolinic acid-treated rat brain homogenates. Quinolinic acid acts through the NMDA subtype of glutamate receptors, and it evokes glutamate-type excitotoxicity [294]. In further studies, chlorogenic acid protected primary cortical neurons from glutamate-induced injury. Importantly, glutamate-induced excitotoxic insult causes an elevation in the concentration of cytosolic Ca^2+^ and chlorogenic acid attenuated the increase in the intracellular Ca^2+^ level [295,296]. In the study by Rebai et al. [296], the neuroprotective effect of chlorogenic acid was mediated by suppressing the accumulation of ROS, restoring the mitochondrial membrane potential, and increasing superoxide dismutase (SOD) activity. Chlorogenic acid also reduced apoptosis by suppressing activation of pro-caspases (i.e., caspase 1, 8, and 9) and calpain. Moreover, it has been proposed that the protein kinase C signaling pathways may be involved in the protective effect of chlorogenic against glutamate-induced neurotoxicity [296]. In another study, chlorogenic acid prevented the AMPA-mediated excitotoxicity in optic nerve oligodendrocytes by inhibiting ROS formation and activation of the antioxidant enzymatic system through the protein kinase C-dependent pathway as well as by the anti-apoptotic caspase and calpain-dependent targets [297].

Several studies focused on the protective effects of chlorogenic acid against the neurotoxicity caused by exposure to Aβ peptide. For example, it displayed significant protective effects towards Aβ_25–35_-induced neuronal damage in PC12 cells as well as in neuroblastoma SH-SY5Y cells [298,299]. In addition, chlorogenic acid suppressed the Aβ_1–42_ self-induced aggregation in PC12 cells [300]. It was also a potent inhibitor of Aβ_1–40_ fibrillization in the ThT assay but it did not inhibit the oligomerization of Aβ_1–42_, which suggests that its interaction with monomeric/oligomeric Aβ proteins differs from the interaction with larger Aβ aggregates [301]. Importantly, chlorogenic acid significantly inhibited Aβ_25–35_-induced autophagy in SH-SY5Y cells by modulating lysosomal function. In the same study, it elevated protein levels of p-mTOR, p-p70s6k and nuclear transcription factor EB (TFEB) indicating that it may enhance the autophagic flux in Aβ2_5-35_-treated SH-SY5Y cells via the regulation of the mTOR/TFEB signaling pathway [299].

The cholinergic deficit in Alzheimer’s disease is a well-known phenomenon, and the restoration of cholinergic function by inhibiting the (acetylcholinesterase) AChE and butyrylcholinesterase (BChE) activity is an effective treatment strategy for Alzheimer’s disease. Given that chlorogenic acid has emerged as a promising neuroprotective agent, its ability to inhibit AChE and BChE activity has also been evaluated. In in vitro studies, it significantly inhibited AChE activity in mouse brain homogenates [302] and in primary hippocampal neuronal cells [287] as well as both AChE and BChE activities in rat brain homogenates [290]. Its inhibitory activity towards AChE and BChE was also demonstrated by using the spectrophotometric Ellman assay [303,304]. Importantly, the anti-AChE [302,304] and anti-BChE [304] activity of chlorogenic was also confirmed in in vivo models of scopolamine-induced amnesia in mice.

In in vitro model of Parkinson’s disease, the impaired viability and enhanced apoptosis of 6-OHDA-damaged SH-SY5Y cells were significantly attenuated by chlorogenic acid pretreatment [305,306]. Chlorogenic acid also suppressed the 6-OHDA-induced ROS production and endoplasmic reticulum (ER) stress in SH-SY5Y cells [305]. Its protective effects against the 6-OHDA-induced toxicity were also reported in the mouse nerve growth factor (mNGF)-differentiated PC12 cells. It prevented cell damage by reducing the 6-OHDA-induced increase in intracellular Ca^2+^ level, suppressing ROS production and inhibiting caspase 3 and 9 activities [307]. Additionally, chlorogenic acid produced a cytoprotective effect against α-synuclein-induced toxicity in catecholaminergic PC12 cells [308] and inhibited α-synuclein fibril assembly [309].

In vivo preclinical studies also provide substantial evidence on the neuroprotective effects of chlorogenic acid. For instance, Vardi et al. [310] demonstrated that chlorogenic acid protected the rat brain cerebellum from oxidative damage induced by methotrexate—a chemotherapeutic agent with severe neurotoxic effects. A 24-day treatment with chlorogenic acid significantly reduced Purkinje cell injury, prevented the methotrexate-induced increase in MDA level as well as decrease in SOD and catalase activity, and reduced glutathione (GSH) content in the cerebellum. In rats with cadmium-induced oxidative brain damage, chlorogenic acid inhibited lipid peroxidation, augmented the antioxidant defense system, and prevented mitochondrial dysfunction and DNA fragmentation [311]. The antioxidant activity of chlorogenic acid also contributed to its protective effect against scopolamine-induced amnesia in mice [302,304]. Acute administration of chlorogenic acid significantly attenuated learning and short-term and long-term memory impairments caused by scopolamine injection in mice. The effect was accompanied by decreased MDA level and increased AChE activity in the hippocampus and frontal cortex [302]. Likewise, repeated administration of chlorogenic acid attenuated the scopolamine-induced learning and memory decline. It also decreased AChE and BChE activities as well as free radical production in the cortex and hippocampus of scopolamine-treated mice [304]. An interesting observation was made by Guo and Li [312] who reported the protective effect of chlorogenic acid against alcohol-induced brain damage in neonatal rats. Treatment with chlorogenic acid attenuated the altered cognitive function in ethanol-exposed pups. In the cerebral cortex and hippocampus, it decreased AChE and caspase-3 activity, reduced MDA and nitrite levels, increased SOD and catalase activity, reduced TNF-α and IL-1β levels, and decreased the level of transcription factor p65 of NF-kB. Thus, the chlorogenic acid protected neonatal rats from ethanol-induced brain damage by decreasing oxidative stress, inflammation, and apoptosis of neuronal cells. In the study by Alarcón-Herrera et al. [313], chlorogenic acid ameliorated the 3-nitropropionic acid-induced toxicity and genotoxicity in mice suggesting its potential protective effect in Huntington’s disease.

Chlorogenic acid was also reported to ameliorate brain ischemia-induced injury in rodents. In models of cerebral ischemia/reperfusion injury, it significantly reduced mortality [314], improved neurological deficit scores [314,315], attenuated sensory-motor functional deficits [316], reduced infarct volume [314,315,316,317], suppressed CA1 pyramidal cell loss [318,319,320], decreased brain edema [315,316,317], and attenuated blood–brain barrier (BBB) damage [316,317]. Importantly, it was demonstrated that chlorogenic acid has a neuroprotective effect against ischemia-induced cognitive deficits. It attenuated learning and memory impairments in ischemic rats [315,319] and in Mongolian gerbils [320]. The protective effect of chlorogenic acid against ischemia-induced brain injury appears to be related with its ability to reduce oxidative stress, neuroinflammation, and cell apoptosis. In rats with cerebral ischemia/reperfusion injury, chlorogenic acid dose-dependently increased the activity of SOD and GSH and suppressed ROS production, lactate dehydrogenase (LDH) release, and MDA accumulation as well as promoted the expression of Nrf2, NQO-1 and heme oxygenase 1 (HO-1) [315]. Likewise, it reduced ROS production and increased SOD2 expression in the CA1 hippocampal region of gerbils with transient global cerebral ischemia [320]. Overexpression of SOD2 (but not SOD1) was also observed in ischemic rats treated with chlorogenic acid [319]. Furthermore, chlorogenic acid suppressed the ischemia-induced increase in pro-inflammatory cytokines, i.e., TNF-α [317,320] and IL-2 [320], as well as overexpression of anti-inflammatory cytokines IL-4 and IL-13 [320]. It also downregulated the expression of an apoptotic marker–caspase-3 [315,317] and increased the expression of an anti-apoptotic protein–Bcl2 in ischemic animals [319]. Moreover, it promoted BDNF [315] and NGF [314,315] expression in the brain of rats subjected to cerebral ischemia/reperfusion. Interestingly, chlorogenic acid was shown to downregulate matrix metalloproteinases (i.e., MMP-2 and MMP-9) mRNA and protein expression in the brain of ischemic rats and to inhibit MMP-2 and MMP-9 activity in in vitro zymography assays. Since extracellular matrix is involved in maintaining the integrity of the BBB and MMP-2 and MMP-9 degrade the extracellular matrix, it seems that the protective effect of chlorogenic acid on BBB damage may result from its ability to reduce expression and activity of MMP-2 and MMP-9 [316]. Interestingly, chlorogenic acid also increased the expression of CD31 (an endothelial marker) and decreased the expression of endothelin-1 in rats with global ischemia, which suggests that it may improve the vascular response by repairing the ischemia-induced endothelial cell damage [319].

Only few studies aimed to evaluate the potential beneficial effects of chlorogenic acid in animal models of Parkinson’s disease. Shan et al. [305] showed that chlorogenic acid attenuated the 6-OHDA-induced Parkinson’s-like behavioral impairments in rats and suppressed the 6-OHDA-induced decrease in striatal dopamine concentration. It also prevented α-synuclein accumulation, increased SOD and glutathione peroxidase (GSH-Px) activities, and restored Bcl-2/Bax expression in the striatum [305]. In rotenone-injected mice, chlorogenic acid ameliorated degeneration of dopaminergic neurons in the substantia nigra and upregulated the antioxidative molecules–metallothionein-1 and 2, in striatal astrocytes [321]. In the study by Singh et al. [322], chlorogenic acid improved motor coordination and neurobehavioral activity in the MPTP-induced model of Parkinson’s disease in mice. Of note, the behavioral effects were accompanied by reduced degeneration of dopaminergic neurons in the substantia nigra. Moreover, chlorogenic acid improved mitochondrial function, suppressed ROS generation, increased SOD and mitochondrial GSH activity, inhibited activation of proapoptotic proteins (Bax and caspase-3), and elevated expression of the anti-apoptotic protein (Bcl2). Since it improved the phosphorylation state of Akt, ERK1/2, and GSK3β, it appears that the neuroprotective effects of chlorogenic acid against MPTP-induced neurotoxicity are mediated, at least in part, by the GSK3β phosphorylation-associated Akt/ERK pathway [322]. It is also worth noticing that chlorogenic acid attenuated the extensive release of release of TNF-α and IL-1β in the substantia nigra of the LPS-injected mice suggesting that this compound may suppress inflammatory response or damage in neurodegenerative diseases including Parkinson’s disease [323].

Two in vivo studies focused on neuroprotective effects of chlorogenic acid against excitotoxicity. In the kainic acid-induced neurotoxicity model in mice, repeated administration of chlorogenic alleviated learning and memory impairments and protected the nNOS-positive neurons in the hippocampal CA1-4 regions from kainic acid-induced injury [324]. Chlorogenic acid also attenuated neuronal loss in the hippocampal CA1 region and produced an anticonvulsant-like effect in the pilocarpine-induced seizure model in mice. In pilocarpine-injected mice, it restored glutamate and gamma-aminobutyric acid (GABA) levels, and decreased NMDA, mGluR1, and mGluR5 receptors expression, which could contribute to the anticonvulsant and neuroprotective effect. Chlorogenic acid also protected from the pilocarpine-induced oxidative stress [325].

Recently, chlorogenic acid has been reported to produce beneficial effects in the APP/PS2 transgenic mice [299]. These double transgenic mice overexpress mutant forms of human APP and human PS2. The APP/PS2 mice display Alzheimer’s-like impairments, e.g., cognitive dysfunction, amyloidosis, inflammation, and impaired synaptic plasticity [326]. Prolonged (180 days) treatment with chlorogenic acid significantly improved spatial memory, decreased neuronal damage in the hippocampus, and suppressed the excessive autophagy in the APP/PS2 mice. It was suggested that neuroprotective effect was likely related with modulation of the mTOR/TFEB signaling pathway [299]. It is noteworthy that cognitive dysfunctions in APP/PS2 mice were also prevented by chronic treatment with coffee polyphenols (including chlorogenic acid). The polyphenols also reduced Aβ plaque deposition in the hippocampus [326].

Summary of in vivo studies on the neuroprotective effects of chlorogenic acid is introduced in Table 4.

*Clinical studies*. While numerous preclinical in vitro and in vivo experiments have been designed to evaluate the neuroprotective effects of chlorogenic acid, only few studies on this matter have been performed in human subjects. Cropley et al. [327] investigated the acute effects of caffeinated coffee, decaffeinated coffee with regular chlorogenic acid content (224 mg), and decaffeinated coffee with higher chlorogenic acid content (521 mg/kg) on cognitive processes and mood in a randomized, double-blind, crossover study with 39 healthy older volunteers. Compared to regular decaffeinated coffee, the chlorogenic acid-rich coffee produced positive effects on mood and mood-related processes. Specifically, it increased alertness, decreased mental fatigue, and alleviated headaches. However, it did not produce substantial pro-cognitive effects. In another randomized placebo-controlled trial, 60 healthy older participants received 6 g of a decaffeinated green coffee blend or 540 mg pure chlorogenic acids or placebo. Cognitive measures were made at 40 and 120 min post-intake. Pure chlorogenic acid did not produce any significant improvement in cognition function when compared to placebo. On the contrary, there was a trend towards chlorogenic acid consumption being associated with slower reaction time and slower information processing speed in comparison to placebo. Decaffeinated green coffee blend improved sustained attention, decision time, and alertness. In addition, both pure chlorogenic acid and the decaffeinated green coffee blend significantly improved symptoms of headache [328]. Despite the lack of significant pro-cognitive effects after single administration [327,328], chlorogenic acid was reported to increase cognitive function following regular prolonged intake [329,330]. Saitou et al. [330] investigated the effects of a 16-week intake of chlorogenic acid-added beverage or placebo on cognitive functions in 38 healthy volunteers (aged 50–69 years) with subjective memory complaints. The obtained results showed that chlorogenic acid improves some cognitive functions (i.e., motor speed, executive function, psychomotor speed, and attention shifting) suggesting that its regular intake may increase individuals’ ability to perform complex tasks by improving both motor activity and cognitive functions. Importantly, blood analysis showed increased levels of apolipoprotein A1 and transthyretin, which are considered biomarkers for the early-stage cognitive decline [330]. Similar effects were observed in the pilot study by Kato et al. [331], who reported that a 6-month intake of chlorogenic acid (330 mg) improved composite and verbal memory, cognitive flexibility, complex attention, executive function, and motor speed in 8 participants with complaints of subjective memory loss. Moreover, biochemical studies revealed decreased plasma Aβ_42_ and Aβ_42_/Aβ_40_ levels and elevated dehydroepiandrosterone sulfate level [331]. In a recent randomized controlled crossover trial, the effect of prolonged chlorogenic acids intake on cognitive function in mild cognitive impairment was investigated [329]. The study was performed on 34 individuals and comprised two 12-week chlorogenic acids intake periods (553.6 mg of chlorogenic acids or placebo twice daily) with a 4-week washout period between them. The cognitive function tests showed that the continuous intake of chlorogenic acids improved cognitive functions in patients with mild cognitive impairment, especially attention and executive function. Taken together, clinical data on the neuroprotective properties of chlorogenic acid are limited. However, some initial evidence suggests that its regular intake may have beneficial effects on cognition function.

Taken together, emerging evidence, from both in vitro and in vivo studies, demonstrates neuroprotective effects of chlorogenic acid. It protects neurons from a wide range of stressors and cell death-inducing agents by ameliorating oxidative stress and neuroinflammation as well as by inhibiting apoptosis and autophagy. In addition, it possesses anti-amyloidogenic effects and inhibits AChE activity. Several signaling pathways, many of which are interdependent, have been proposed to be involved in the neuroprotective effects of chlorogenic acid. No differences between neuroprotective effects of caffeinated and decaffeinated coffee suggest that chlorogenic acid, the most abundant active coffee compound, may significantly contribute to the beneficial effects of coffee on some neurodegenerative disease and cognitive decline. A few preliminary clinical trials [327,329,330,331] showed that regular, but not acute, chlorogenic acid intake improves cognitive function in humans. Therefore, large-scale longitudinal clinical studies are highly warranted to provide more insight into the beneficial effects of chlorogenic acid in neurodegenerative diseases. Further studies are also required to better characterize the pharmacokinetics and metabolism of chlorogenic acid in humans and to identify its potential adverse effects.

### 4.3. Neuroprotective Effects of Caffeic Acid

Caffeic acid is produced by many plant species, not only by Coffea sp. Like many other polyphenols, caffeic acid exerts potent antioxidant and free radical scavenging properties [332]. Its antioxidant activity appears to be greater than the antioxidant activity of many other important coffee components including chlorogenic acid [333]. Moreover, numerous studies showed that caffeic acid has anti-inflammatory, anti-mutagenic, antibacterial, and anti-carcinogenic properties, which could be linked to its high antioxidant activity [334,335]. There is also a growing body of evidence showing possible neuroprotective effects of caffeic acid. It is noteworthy that its naturally occurring derivative–caffeic acid phenyl ester (CAPE) has also been extensively studied for neuroprotective properties. Chemical versatility and modifiability of caffeic acid caused its phenylpropanoid scaffold to become a commonly used template for the development of new derivatives with enhanced pharmacokinetic properties, increased bioactivity, and better safety profile [336].

*Preclinical studies*. Numerous in vitro studies have demonstrated that caffeic acid displays a broad-spectrum neuroprotective profile. Several reports showed that caffeic acid is protective against the H_2_O_2_-induced oxidative stress. For example, it attenuated the H_2_O_2_-induced cell injury in cultured cerebellar granule neurons [291], PC12 cells [337,338,339], neuroblastoma SH-SY5Y cells [340], and rat cortical slices [285]. Caffeic acid also suppressed intracellular ROS accumulation as well as the release of LDH from PC12 cells exposed to H_2_O_2_ [337,338]. Oboh et al. [290] reported that caffeic acid dose-dependently inhibited the excessive MDA production in rat brain homogenates following incubation with another pro-oxidant agents—FeSO_4_ and SNP (a NO donor). Its beneficial effects against the NO-induced neurotoxicity were also reported by Taram et al. [291] who showed that caffeic acid protects cerebellar granule neurons from the SNP-induced death. The effect was accompanied by reduced NO production indicating that caffeic acid protects neurons against nitrosative stress via free radical scavenging activity. In the same study, caffeic acid provided significant protection against the glutamate/glycine-induced neurotoxicity, which is in line with previous reports showing that this compound protects primary cultures of rat cortical neurons from the excitoxicity induced by glutamate [296,341,342]. It is noteworthy that the neuroprotective effect was mediated by inhibition of the glutamate-induced intracellular Ca^2+^ influx and subsequent reduction in ROS formation [342]. Caffeic acid also exhibited anti-apoptotic properties by suppressing the glutamate-induced caspase activation [296]. Moreover, it ameliorated (via inhibiting 5-LOX activation) the NMDA-induced early and delayed injuries in PC12 cells [343] and the quinolinic acid-induced oxidative stress in rat brain homogenates [290] and rat striatal slices [344]. Interestingly, caffeic acid also attenuated cerebellar granule neurons death induced by brefeldin A–an ER stressor [291]. In contrast, it did not inhibit SH-SY5Y cell death induced by another ER stressor—tunicamycin [345]. This could have been due to the fact that these two agents have distinct mechanisms of action. Brefeldin A inhibits transport between the ER and Golgi apparatus, whereas tunicamycin suppresses protein glycosylation in the Golgi apparatus [291,345]. Furthermore, caffeic acid displayed protective activity against caspase-dependent intrinsic apoptosis in cerebellar granule neurons [291]. It seems that the anti-apoptotic effect of caffeic acid may result from its ability to modulate the anti-apoptotic and pro-survival pathways in neuronal cells. For instance, it upregulated anti-apoptotic proteins (Bcl2 and Bcl-XL) and downregulated pro-apoptotic proteins (Bad, PARP, and cleaved caspase 3) in mouse retinal ganglion cells subjected to the hypoxia-induced damage. In HT22 mouse hippocampal cells, caffeic acid reduced the acrolein-induced neurotoxicity by activation of the pro-survival Akt/GSK3β signaling pathway [346]. It is noteworthy that it also protected cerebellar granule neurons from death evoked by PS-341—a proteasome inhibitor. Inhibition of proteasome activity induces cell apoptosis by accumulation of c-Jun and a pro-apoptotic Bim protein [291]. Since caffeic acid was shown to activate the AKT signaling that promotes cellular survival via inhibition of Bim protein [346], it seems that this compound confers neuroprotection against PS-341 by inhibition of the pro-apoptotic Bim protein. Finally, caffeic acid ameliorated the levodopa-induced toxicity in neuroblastoma SH-SY5Y cells [347] and the Aβ-induced neurotoxicity, by the inhibition of calcium influx and tau phosphorylation, in PC12 cells [348].

Animal studies have provided further support for neuroprotective effects of caffeic acid. Yang et al. [349] showed that repeated administration of caffeic acid protected mouse brain from the aluminum-induced damage. It reversed the learning and memory impairments caused by aluminum overload and antagonized the aluminum-induced increase in brain MDA levels and decrease in the expression of choline acetyltransferase. It also decreased overexpression of APP, Aβ, and 5-LOX. Likewise, caffeic acid improved the learning and memory deficits in the aluminum-treated rats and reduced the aluminum-induced increase in AChE, catalase, and glutathione-S-transferase activity (GST) as well as GSH and nitrate levels in the brain [350]. Similar results were obtained by Deshmukh et al. [351] who reported that caffeic acid ameliorated the streptozotocin-induced neurocognitive deficits. It improved non-spatial memory performance in the object recognition task and spatial memory performance in the Morris water maze test. Moreover, it attenuated streptozotocin-induced oxidative stress and produced dose dependent decrease in AChE activity. Decreased brain AChE activity was also observed in the Aβ_1–40_-induced neurotoxicity in rats [352]. Interestingly, a 30-day treatment with caffeic acid improved the learning and memory abilities in naïve rats and inhibited significantly the AChE activity in the cerebral cortex and the striatum but increased the AChE activity in the hippocampus, hypothalamus, and pons [353]. However, data from in vitro studies on the possible anti-AChE activity of caffeic acid are inconsistent. Oboh et al. [290] reported that this compound inhibited both the AChE and BChE activity in rat whole brain homogenates. In other studies, caffeic acid exhibited AChE inhibitory effect in the cerebral cortex of rat brain, whole brain without the cerebral cortex [354], and whole brain with the cerebral cortex [350]. In contrast, Anwar et al. [353] reported that caffeic acid significantly increased the AChE activity in the cerebral cortex, cerebellum, and hypothalamus, while in the striatum, hippocampus, and pons, it did not alter the enzyme activity. This suggests that caffeic acid may have the specific selectivity in relation to the AChE from different brain regions [353].

Neurodegeneration is also a hallmark feature of epilepsy. There are only few reports on the neuroprotective effects of caffeic acid in animal models of seizure and epilepsy. It produced an anticonvulsant-like effect in the pilocarpine-induced seizure model in rats and decreased hippocampal damage caused by seizures. Moreover, it decreased lipid peroxidation level and nitrite content and increased SOD and catalase activity in the hippocampus following seizures [355]. In addition, caffeic acid prevented the quinolinic acid-induced behavioral alterations in rats [344,356] and restored the redox status in rat striatum by increasing the levels of GSH and GSH/GSSG, reversing the rise in oxidized glutathione level in quinolinic acid-treated animals [356], which add support to the neuroprotective properties of this coffee compound against the excitotoxic damage. In the kainic acid-induced excitotoxicity model in rats, caffeic acid prolonged the latency to seizures and reduced neuronal loss in the CA3 hippocampal field [357]. Further studies, however, did not confirm the anticonvulsant-like properties of caffeic acid. It was not effective against the pentylenetetrazole- and pilocarpine-induced seizures in mice [358] and did not produce antiepileptogenic effect in the kindling model of epilepsy [359]. Nonetheless, caffeic acid presented neuroprotective effect against the pilocarpine-induced genotoxic damage in the mouse hippocampus [358]. It also showed neuroprotective action against DNA damage and oxidative stress in the cerebral cortex caused by the pentylenetetrazole-induced kindling in mice [359].

Several reports demonstrated that caffeic acid has protective effects on focal [360,361,362,363] and global [364] cerebral ischemia/reperfusion injury in rodents. Caffeic acid significantly reduced infarct volume and improved neurological deficit scores in mice [361] and rats [362,363] after induction of focal cerebral ischemia. It also decreased cell damage in the ischemic hippocampal CA1 region of Mongolian gerbils [360] and attenuated hippocampal neurons injury induced by global cerebral ischemia-reperfusion in rats [364]. Moreover, Pinheiro Fernandes et al. [361] showed that caffeic acid protects against ischemia-induced cognitive impairments. It attenuated working, spatial, and long-term aversive memory deficits in mice with focal cerebral ischemia. A beneficial effect of caffeic acid on cognitive decline following ischemia was also reported by Liang et al. [364]. In rats with global cerebral ischemia, it attenuated learning and memory deficits. There is evidence of microglia activation in ischemic stroke, and it appears that the neuroprotective effects of caffeic acid against ischemic injury may result, at least in part, from its ability to attenuate astrocyte proliferation and microglia activation. It was demonstrated that caffeic acid inhibited astrocyte proliferation 14 days after focal cerebral ischemia in rats [363] and decreased microglia activation and its protein level in ischemic gerbils [360]. The protective effects of caffeic acid in ischemia models may be also related to its ability to inhibit 5-LOX activity as it suppressed the production of leukotrienes (i.e., 5-LOX metabolites) in the rat brain after focal ischemia induction [363] as well as in the PC12 cells exposed to oxygen-glucose deprivation/reperfusion (OGD/OGD-R) insult—an in vitro model of ischemia/reperfusion [362]. Furthermore, caffeic acid downregulated the 5-LOX mRNA and protein overexpression in rats with global cerebral ischemia-reperfusion injury [364]. A declined expression of 5-LOX after caffeic acid treatment was also observed in rats with focal cerebral ischemia [362]. In OGD/OGD-R PC12 cells, caffeic acid suppressed the production of arachidonic acid by lipoxygenase metabolism, maintained the ultrastructure and integrated function of mitochondria, decreased ROS generation, and finally protects the cells from ischemia [362]. It is also worth mentioning that caffeic acid decreased caspase 3 immunoreactivity [361], reduced NF-κBp65 overexpression, decreased the brain MDA level and increased SOD activity [364], which further suggests that it may also ameliorate inflammation and oxidative stress following global cerebral ischemia-reperfusion injury. Interestingly, caffeic acid was also shown to inhibit the reduction of synaptophysin expression after ischemic insult in mice. Of note, synaptophysin is a membrane-associated protein that is an important marker of synaptogenesis, synaptic density, and neural development. Its expression decreases following ischemia, which is correlated with memory deficits [361].

Caffeic acid attenuated the lesion and neuron loss after cryoinjury in mice, which suggests its neuroprotective effect against traumatic brain injury. It inhibited astrocytes activation and thereby attenuating their proliferation and glial scar formation in the late phase of cryoinjury. Moreover, it inhibited the decrease in SOD activity and the increase in MDA content in the brain after cryoinjury [365]. In an in vivo model of Alzheimer’s disease, it ameliorated the Aβ_1–40_-induced learning and memory impairment, increased synaptophysin expression and weakened the cerebral damage in rats. The effect was accompanied by inhibition of AChE activity, suppression of oxidative stress and reduced inflammation [352].

It was showed that caffeic acid may be also a preventive agent against the progression of Parkinson’s disease. In vitro, caffeic acid provided protection against the 5-S-cysteinyl-dopamine-induced neurotoxicity in mouse cortical neurons [366]. Li et al. [367] showed that this compound protects against dopaminergic neurodegeneration in in vivo model. In the LPS-treated rats, it attenuated the loss of nigral dopaminergic neurons and microglia activation [367]. Next studies showed that caffeic acid reversed the paraquat-induced movement impairment (i.e., climbing capability) in Drosophila melanogaster–a valid model of Parkinson’s disease [368]. In the same model, caffeic acid reduced fly mortality, restored mitochondrial activity, and attenuated the paraquat-induced oxidative stress [369]. Moreover, Tsai et al. [370] reported the neuroprotective effect of this compound in the MPTP mouse model of Parkinson’s disease. It decreased the MPTP-caused inflammatory stress by suppressing the production of inflammatory cytokines (i.e., IL-1β, IL-6, TNFα, IL-4 and IL-10), lowering the production of NO and prostaglandin E2, and the activity of total NOS and COX-2. Caffeic acid intake also declined the expression of iNOS, nNOS, and COX-2 as well as retained the expression and production of BDNF, GDNF, and tyrosine hydroxylase in the striatum of the MPTP-treated mice. Although caffeic acid failed to affect dopamine transporter expression, it restored dopamine, DOPAC and HVA levels [370]. In rotenone-injected mice, chlorogenic acid attenuated degeneration of dopaminergic neurons in the substantia nigra and increased the expression of metallothionein-1 and 2 in striatal astrocytes [321]. In another study, caffeic acid produced neuroprotective effects in the α-synuclein-induced models of Parkinson’s disease. α-Synuclein is a presynaptic neuronal protein that is implicated in the pathophysiology of this disease. In SH-SY5Y cells overexpressing A53T α-synuclein, caffeic acid alleviated the cell damage caused by overexpression of A53T α-synuclein, suppressed the accumulation of A53T α-synuclein, and induced the JNK/Bcl-2-mediated cell autophagy to degrade A53T α-synuclein. In next experiments, caffeic acid administered for 8 weeks alleviated motor deficits, induced autophagy, decreased the accumulation of A53T α-synuclein, and ameliorated the loss of dopaminergic neurons in the substantia nigra of A53T transgenic mice (i.e., mice expressing the human A53T mutant of α-synuclein) [371]. Recently, caffeic acid was also reported to improve survival and motor performance in wild type *Caenorhabditis elegans* exposed to dopaminergic toxin 6-OHDA, which was in line with data obtained from in vitro studies. Specifically, caffeic acid prevented the loss of reductive capacity, cell damage, and the oxidative damage induced by 6-OHDA in rat cortical slices. Additionally, similar neuroprotective effects of caffeic acid were observed in both *Caenorhabditis elegans* and rat cortical slices treated with FeSO_4_ and quinolinic acid. Based on further molecular studies, it was concluded that caffeic acid confers neuroprotection against different toxic insults via the Nrf2/ARE pathway in the mammalian cortical tissue and the orthologous skn-1 pathway in the worms [372].

CAPE, a caffeic acid derivative, was also reported to exhibit neuroprotective effects in numerous in vitro studies. For example, it prevented the glutamate-induced excitotoxicity by inhibiting phosphorylation of p38 and caspase-3 activation in cerebellar granule neurons [373]. CAPE also protected T22 mouse hippocampal cells from acrolein-induced neurodegeneration through modulating MAPKs and Akt/GSK3b signaling pathways [346]. Moreover, it was shown to be a potent inducer of HO-1 in astroglial cells and neurons [374]. Interestingly, inhibition of NF-kB by CAPE downregulated the release of pro-inflammatory miRNAs from primary human neuronal–glial cells stressed with the brain tissue-derived extracellular fluid from patients with Alzheimer’s disease [375]. In an animal model of Alzheimer’s disease, CAPE decreased Aβ_1–42_–induced neuronal apoptosis and neuroinflammation and improved learning and memory [376]. Furthermore, CAPE was effective against the MPP^+^- [377,378] and 6-OHDA-induced [379,380] neurotoxicity in vitro and attenuated the dopaminergic neuronal loss induced by 6-OHDA in mice [381] and rats [382] as well as by MPTP in mice [378], which makes it a potential therapeutic candidate for the prevention and/or treatment of Parkinson’s disease. CAPE was also reported to produce neuroprotective effects in animal models of ischemia. It reduced focal cerebral ischemia injury in both mice and rats possibly through its antioxidant and anti-inflammatory effects and/or via the upregulation of NO production [383,384,385]. In addition, it inhibited apoptotic cell death in ischemic rats by downregulating caspase 3 and upregulating anti-apoptotic protein Bcl-xL [385]. CAPE also exhibited a preventive effect on early brain injury after subarachnoid hemorrhage in rats [386]. In other studies, this compound reversed cognitive impairment induced by streptozotocin [387], d-galactose [388], and cadmium [389].

Taken together, in vitro studies show that caffeic acid protects neurons from a wide range of cell death-inducing agents. Moreover, data from animal studies (Table 5) indicate that this compound may prevent neuronal damage/death caused by different stressors suggesting that caffeic acid is a promising neuroprotective compound for the prevention and treatment of neurodegenerative diseases. Unfortunately, there are no human intervention studies or clinical trials on this matter. Nevertheless, based on the above-mentioned reports, it appears that the neuroprotective properties of coffee may be largely attributed to the presence of caffeic acid.

### 4.4. Neuroprotective Effects of Trigonelline

Trigonelline, the second most abundant alkaloid in coffee beans, exerts a wide range of pharmacological effects including an anti-hyperglycemic, anti-hyperlipidemic, antibacterial, antiviral, and anti-tumor activity [390,391]. In contrast to caffeine or chlorogenic acid, neuroprotective effects of trigonelline have not been so extensively studied. However, there are several preliminary in vitro and in vivo studies showing that trigonelline provides neuroprotection and may be beneficial in the management of some neurodegenerative conditions. Few reports focused on the possible protective effects of trigonelline against Alzheimer’s disease. Molecular docking study showed that it has high affinity to the Aβ_1-42_ peptide altering its structure and thereby inhibiting its aggregation [392]. In rat cortical neurons, trigonelline prevented dendritic and axonal atrophy induced by administration of Aβ_25–35_. It also reversed the Aβ_25–35_-induced impairment of spatial memory in mice [393]. Moreover, trigonelline produced neuroprotective effect in a rat model of Alzheimer’s disease induced by administration of Aβ_1–40_. Pretreatment of Aβ_1–40_-microinjected rats with trigonelline significantly improved spatial recognition memory in the Y maze test and performance in the novel object recognition task. Importantly, histological analysis showed that trigonelline prevented Aβ_1–40_-induced loss of hippocampal CA1 neurons. Furthermore, it decreased oxidative stress parameters; augmented antioxidant defensive system; reduced hippocampal levels of glial fibrillary acidic protein (GFAP), S100b, COX-2, TNF-α, and IL-6; and improved mitochondrial membrane potential. Thus, it appears that the neuroprotective effect could be mediated by the reduction of oxidative stress, neuroinflammation, astrocyte activity, and preservation of mitochondrial integrity [394]. In another study, trigonelline ameliorated LPS-induced cognitive decline in mice in the Morris water maze task and Y maze test, which suggests that it can improve both spatial and working memory. The behavioral effects were accompanied with reduced oxidative stress parameters, decreased level of pro-inflammatory cytokines, decreased AChE activity, and upregulated BDNF level in both the hippocampus and cortex [395]. Similar results were obtained by Khalili et al. [396] who reported that trigonelline diminished the LPS-induced learning and memory disturbances via suppression of hippocampal oxidative stress, neuroinflammation, and AChE activity. In addition, they showed that the anti-inflammatory effect of trigonelline could be mediated by the NF-κB and TLR4 signaling pathways.

It appears that trigonelline may also produce neuroprotective effects due to its antiglycating properties. In in vitro experiments, it suppressed formation of advanced glycation end products (AGEs), pentosidine compounds, and Amadori compounds (i.e., early markers of protein glycation). This is an important observation as AGEs contribute to amyloidosis in Alzheimer’s disease suggesting that glycoxidation plays a crucial role in the pathogenesis of this disease. It was demonstrated that chronic administration of d-galactose impairs learning and memory, induces oxidative damage, elevates the AGEs levels, and increases AChE activity in mice. It is noteworthy that trigonelline treatment significantly improved cognitive performance in the Morris water maze and Y-maze tests, reduced oxidative stress, and decreased AGEs and AChE levels in d-galactose-treated animals [397].

Neuroprotective properties of trigonelline were also reported in experimental models of Parkinson’s disease. In unilaterally 6-OHDA-lesioned rats, it reduced apomorphine-induced rotations, increased the viability of neurons in the substantia nigra pars compacta, prevented apoptosis, and restored the MDA level [398]. Gaur et al. [399] showed however that trigonelline (but only at low doses) increased the number of ipsilateral rotations in the 6-OHDA-lesioned rats, indicating dopamine releasing action. In the same study, trigonelline pretreatment also reversed the MPTP-induced motor dysfunctions in mice. Additionally, it was demonstrated that this coffee compound is devoid of anticholinergic effects and does not inhibit MAO-B activity [399].

It is also worth mentioning that trigonelline was neuroprotective in ischemic stroke [400] and oxygen-glucose deprivation-induced neural injury [391]. Trigonelline injected immediately following ischemia induction produced neuroprotection in rats by reducing cerebral infarct, which was accompanied with improvement in motor and neurodeficit scores. Moreover, it reduced the glutathione-mediated expression of myeloperoxidase in the cortical brain region and augmented the antioxidant status. Consistent with in vivo findings, trigonelline increased the PC12 cell viability following hypoxia induction in in vitro experiments [400]. Qiu et al. [391] demonstrated that trigonelline protected hippocampal neurons from the oxygen-glucose deprivation/reperfusion-induced injury. It also ameliorated oxidative stress, attenuated inflammatory response, and inhibited cell apoptosis in hippocampal neurons. Of note, the neuroprotective effect was probably mediated by the activation of PI3K/Akt signaling pathway.

Taken together, the above-mentioned reports (Table 6) consistently demonstrated that trigonelline may be a promising neuroprotective agent mainly due to its antioxidant, anti-inflammatory, and anti-apoptotic properties. However, the exact molecular mechanisms underlying the neuroprotective effects of trigonelline need to be established. Some studies showed possible involvement of the TLR4/NF-κB [396] and PI3K/Akt [391] signaling pathways, but these are preliminary findings only. It is noteworthy that a recent study showed trigonelline exerts an antidepressant-like effect in mice via reduction of NMDA receptor activity [401], which deserves further investigation as the NMDA-mediated glutamatergic transmission is also implicated in the pathophysiology of neurodegenerative disorders. It has been postulated that coffee may exert health promoting effects, including neuroprotective ones, via dampening inflammation-induced NF-κB activity and activation of the Nrf2 system with subsequent enhancement of the cell defense response [402,403]. Indeed, several coffee constituents (i.e., chlorogenic acids, caffeic acid, kahweol, and cafestol) have been reported to act as inducers of the Nrf2 pathway. In contrast, trigonelline is a potent inhibitor of the Nrf2 transcription factor and the inhibitory effect is observed at physiologically relevant concentrations. Importantly, roasting of coffee beans increases their ability to activate the Nrf2/ARE pathway. This is related to the formation of new potent activators of the Nrf2 transcription factor during roasting process (e.g., *N*-methylpyridinium ion). A lower content of trigonelline in roasted coffee may also contribute to the stronger activation of Nrf2/ARE pathway [402,403,404]. Thus, the question also arises whether trigonelline significantly contributes to the beneficial effects of coffee beverages consumption in neurodegenerative diseases.

### 4.5. Neuroprotective Effects of Kahweol and Cafestol

Kahweol and cafestol are two coffee-specific diterpenes present in unfiltered coffees such as Scandinavian-style boiled coffee, Turkish-style coffee, French press coffee, and espresso. Although these two compounds are known mainly from their hypercholesterolemic effects, a growing body of evidence shows that kahweol and cafestol also have many beneficial effects such as anti-inflammatory, antioxidant, hepatoprotective, anti-diabetic, and anti-carcinogenic activities [405,406]. However, data on the neuroprotective properties of these two compounds are quite limited.

Kahweol is a potent antioxidant agent with cytoprotective properties [407,408], which suggests that it should also produce neuroprotection. Indeed, Hwang and Jeong [409] demonstrated the protective effect of kahweol against the 6-OHDA-induced oxidative stress in the dopaminergic SH-SY5Y neuronal cells indicating its possible neuroprotective effects in Parkinson’s disease. They showed that kahweol significantly increased cell survival following 6-OHDA treatment and reduced 6-OHDA-induced ROS production. It also induced heme oxygenase-1 expression and Nrf2 nuclear translocation in dopaminergic neuronal cells. Next experiments demonstrated the involvement of PI3K/Akt and p38 signaling in kahweol-induced heme oxygenase-1 upregulation [409]. Kahweol was also protective in the human neuroblastoma SH-SY5Y cells exposed to methylglyoxal. It decreased the methylglyoxal-induced loss of mitochondrial membrane potential, prevented the mitochondria-related bioenergetics decline, and suppressed production of ROS and RNS [407]. Likewise, kahweol promoted mitochondrial protection in SH-SY5Y cells exposed to H_2_O_2_, decreased the level of oxidative stress markers, and reduced the production of ROS [410]. In both of the aforementioned studies, the protective effect of kahweol was mediated via activation of the PI3K/Akt and p38 MAPK/Nrf2 signaling pathways [407,410], which is in line with previous findings by Hwang and Jeong [409]. It is also worth mentioning that kahweol was protective against the H_2_O_2_-induced DNA damage [408]. This is an important observation in view of the fact that oxidative DNA damage is one of the earliest changes in neurodegenerative diseases.

It is noteworthy that there is one in vivo study showing a possible protective effect of kahweol on brain neurons. In mice, acute systemic administration of kahweol ameliorated the traumatic brain injury-induced brain parenchymal damage and reversed short- and long-term functional outcomes. These effects were accompanied by reduced production of cytokines (IL-1β, MIP-1α, MIP-2, and TIMP-1) in the brain, decreased microglia/macrophage activation, and reduced neutrophil and leukocyte infiltration. In addition, continuous administration of kahweol potentiated the protective effects of a single-dosage treatment [411]. This is an important finding as the traumatic brain injury is associated with an increased risk of neurodegenerative diseases, though the mechanism underlying this association is not clear [412].

To date, only one study focused on the possible neuroprotective properties of cafestol. Trinh et al. [413] studied its protective effect in *Drosophila* models of Parkinson’s disease. They showed that cafestol conferred neuroprotection in both α-synuclein transgenic and parkin null mutant flies. Moreover, it was demonstrated that the effect was mediated through the Nrf2-dependent mechanism [413]. Similarly to kahweol, cafestol was also demonstrated to produce antioxidant and anti-inflammatory effects. Other studies showed that cafestol may activate the Nrf2/ARE signaling pathway, increase the expression of HO-1, eliminate excessive ROS production, and protect against oxidative DNA damage [406,408]. All these effects may contribute to its potential neuroprotective action.

Summarizing, there are some initial evidence suggesting neuroprotective effects of kahweol and cafestol. However, the possible protective effects of the two coffee diterpenes have to be confirmed in animal models of neurodegenerative diseases. More insight into the absorption and metabolism of kahweol and cafestolis also needed. A special attention should be given to the ability of kahweol and cafestol (and/or their active metabolites) to cross the BBB as their brain penetration has not been studied so far. Moreover, since the coffee diterpenes are known to raise serum cholesterol level, it will be necessary to carefully evaluate the risk/benefit ratio of using them for neuroprotection.

## 5. Summary and Conclusions

**Figure 3:**
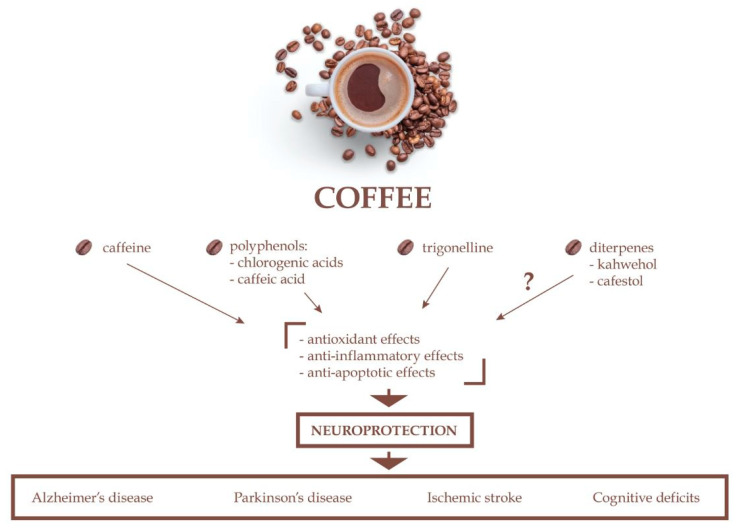
Summary of the neuroprotective effects of coffee.

Extensive in vitro and in vivo studies have demonstrated that coffee and its bioactive compounds exert neuroprotective effects suggesting their preventive and/or therapeutic potential for different neurodegenerative conditions (Figure 3). Among them, caffeine has been the most extensively investigated and the beneficial effects of coffee consumption can be largely (but not solely) attributed to caffeine. However, numerous reports show that other coffee compounds may independently produce neuroprotective effects indicating that decaffeinated coffee could be also very effective in neurodegenerative conditions. Polyphenolic acids (i.e., chlorogenic acids and caffeic acid) and trigonelline appear to be the most promising, but in contrast to caffeine, there is a lack of epidemiological studies or clinical reports on their protective effects in neurodegenerative diseases. There are only preliminary data on the possible beneficial effects of chlorogenic acid on cognitive function in humans. Thus, large-scale observational and clinical studies are highly warranted to provide more insight into the neuroprotective effects of caffeine, coffee polyphenols, and trigonelline. Each compound should be studied separately as each one has its own unique properties and can have different effects depending on the disease. Moreover, the exact mechanism(s) by which each component confers neuroprotection should be elucidated. Their bioavailability and long-term adverse effects also warrant further investigation.

On the other hand, the effects of coffee in neurodegenerative diseases may result from a synergistic action of many active compounds. Therefore, epidemiological and clinical studies should be continued to fully evaluate the association between regular coffee beverage consumption and the risk of neurodegenerative diseases. It should be, however, emphasized that such studies face a variety of challenges, and one of the most important is a high variability in the final composition of coffee beverage that depends on many factors such as coffee beans origin, roasting level, and brewing techniques [182]. In addition, since bioavailability of ingredients may depend on each individual’s metabolism, the response to coffee intake can vary substantially across individuals [183], which should also be taken into account when studying the effect of coffee intake in neurodegenerative diseases.

## Figures and Tables

**Table 1:** The chemical composition of green and roasted coffee beans [17,35,36].

Compounds	% Content in Dry Weight of Coffee Beans	
	Green Coffee	Roasted Coffee
Carbohydrates – polysaccharides—cellulose, arabinogalactan, galactomannan – oligosaccharides—stachyose, raffinose – disaccharides—sucrose – monosaccharides—glucose, galactose, arabinose, fructose, mannose, mannitol, xylose, ribose	60	43
Lipids – triglyceride – sterols—stigmasterol, sitosterol – fatty acids—linoleic, linolenic, oleic, palmitic, stearic, arachidic, lignoceric, behenic acid – fatty acids with pentacyclic – diterpenes—cafestol, kahweol – waxes – tocopherols – phosphatides	8–18	10–15
Proteins – amino acids—asparagines, glutamic acid, alanine, aspartic acid, lysine	9–16	7.5–10
Other nitrogenous compounds – caffeine – trigonelline – nicotinic acid	1–6 0.9–3.33 0.88–3.42 2 × 10^−6^–3 × 10^−6^	1–2 1 0.7–1 0.01–0.04
Melanoidins	–	25
Minerals	4	3.7–5
Organic and inorganic acids and esters – chlorogenic acids – aliphatic acids and quinic acid – other organic and inorganic acids	6–15 4–14.4 0.7–2.5 2	6 1–4 1.4–2.5 <0.3

**Table 2:** The chemical composition of coffee beverages or drew [35].

Compound	Content in Coffee Beverages or Brew Obtained from Blends of Arabica and Robusta Coffee [mg per 100 mL]
Water	94,000–98,500
Aliphatic acids and quinic acid	692–2140
Polysaccharides (galactomannans and type II arabinogalactans)	200–700
Lipids	180–400
Proteins	120–400
Simple saccharides (arabinose, mannose, galactose, sucrose)	0–200
Bioactive ingredients:	
Melanoidins	500–1500
Chlorogenic acids	32–500
Caffeine	50–380
Trigonelline	12–50
Diterpenes (cafestol and kahweol)	0.2–10
*N*-methylpyridinium	2.9–8.7
Serotonin	0–1.4
Polyamines (spermine and spermidine)	0.4
Phenolic substances	0.1–0.2
β-carbolins (norharman and harman)	0.004–0.08
Melatonin	0.006–0.008
*Minerals:*	
Total ashes	150–500
Potassium (K)	115–320
Sodium (Na)	1–14
Phosphorous (P)	3–7
Calcium (Ca)	2–4
Iron (Fe)	0.02–0.13
Manganese (Mn)	0.02–0.05
Zinc (Zn)	0.01–0.05
*Vitamins:*	
B_3_	0.8–10
B_9_	1
C	0.2
B_2_	0.177
K	0.1
E	0.01
B_6_	0.002
B_1_	0.001
*Undesirable substances:*	
Acrylamide	3.9–840
Furan	3.8–262
*N*-alkanoyl-5-hydroxytryptamides	1.2–34.3

**Table 3:** Summary of in vivo studies on the neuroprotective effects of caffeine.

Animals	Treatment	Model	Behavioral Tests	Main Outcomes	Ref.
APPsw transgenic mice (background C57, B6, SJL and Swiss-Webster mice)	0.3 mg/mL caffeinated water beginning at 4 months of age for 4 months (daily dose of 1.5 mg caffeine to each mouse)	Genetic model of Alzheimer’s disease	Open-field test, balance beam test, string-suspension, Y-maze test, elevated plus-maze test, Morris water maze test, circular platform test, platform recognition test, radial arm water maze test	(1) Improvement of cognitive task of spatial learning/reference memory, working memory, and recognition/identification, (2) decrease in Aβ production due to reduced expression of presenilin 1 and β-secretase, (3) restored adenosine levels in the brain to normal	[194]
APPsw transgenic mice (background C57, B6, SJL and Swiss-Webster mice)	0.3 mg/mL caffeinated water beginning at 18–19 months of age for 4–5 weeks (daily dose of 1.5 mg caffeine to each mouse)	Genetic model of Alzheimer’s disease	Open-field test, balance beam test, string-suspension, Y-maze test, elevated plus-maze test, Morris water maze test, circular platform test, platform recognition test, radial arm water maze test	(1) Improvement of superior working memory, (2) reduced Aβ deposition in the hippocampus and entorhinal cortex, (3) decrease in brain soluble Aβ levels, (4) aged APPsw mice exhibited memory restoration and reversal of AD pathology, (5) caffeine suppression of β-secretase involves the cRaf-1/NFκB pathway	[195]
Albino rats (Morini, Wistar derived strain)	15, 45, and 80 mg/kg/day (s.c.) for 15 days	–	Staircase test	No effect on memory retention	[192]
CF1 mice	1 mg/mL for 12 months	–	Object recognition test	(1) Aged mice exhibited lower performance in the recognition memory compared with adults, (2) caffeine-treated mice showed similar performance to adult mice in the object recognition test and an improvement compared with their age-matched control mice, (3) caffeine counteracted the age-related increase in BDNF and TrkB immunocontent	[193]
CF1 mice	chronic (12 days) treatment with caffeine (1 mg/mL, p.o.); subchronic (4 days) treatment with caffeine (30 mg/kg, i.p.); acute caffeine treatment (30 or 80 mg/kg, i.p.) 30 min treatment before Aβ administration	Aβ_25–35_-induced neurotoxicity	Inhibitory avoidance test, Y-maze test	(1) Chronic and subchronic treatment with caffeine prevent Aβ-induced cognitive impairment, (2) A_2A_ receptors are engaged in the control of Aβ-induced cognitive dysfunction	[197]
Wistar rats	30 mg/kg (p.o.) daily per 10 days	Aging	Novel object recognition memory test, open field test	(1) Reversed age-related memory deficit, (2) normalized oxygen and NRS levels increased in brains of aged rats, (3) normalized Na^+^/K^+^-ATPase activity inhibited in brains of aged rats, (4) A_2A_ receptors affect the impact and formation of free radicals in neuronal preparations	[199]
Sprague-Dawley rats	3 mg/kg/day (i.p.) for 60 days	d-Galactose induced neurodegeneration	Y-maze test	(1) Attenuated memory impairment; (2) reduced oxidative stress via the reduction of 8-oxoguanine; (3) suppressed stress kinases p-JNK; (4) reduced d-galactose-induced neuroinflammation through alleviation of COX-2, NOS-2, TNFα, and IL-1β; (5) reduced cytochrome C, Bax/Bcl2 ratio, caspase-9, caspase-3, and PARP-1 levels; (6) prevented neurodegeneration	[191]
THY-Tau22 male mice (C57Bl6/J background)	0.3 mg/mL caffeinated water beginning at 2 months until 12 months of age (daily dose of 1.5 mg caffeine to each mouse)	Genetic model of Alzheimer’s disease	Morris water maze test	(1) Prevented development of spatial memory impairments, (2) reduced tau phosphorylation and proteolytic fragments, (3) modulated hippocampal neuroinflammatory and oxidative stress markers	[200]
Sprague-Dawley rats	0.3 or 0.6 mg/mL caffeinated water for 3 weeks or just once	–	–	Chronic caffeine treatment (1) induced ventriculomegaly, (2) increased production of CSF, which were associated with the enhancement of the expression of Na^+^/K^+^-ATPase and increased CBF	[202]
New Zealand white rabbits	0.5 mg/day or 30 mg/day in the drinking water for 12 weeks	2% cholesterol-enriched diet	–	(1) Decreased cholesterol-enriched diet-induced increase in Aβ production and accumulation, (2) reduced cholesterol-induced increase in tau phosphorylation, (3) attenuated cholesterol-induced increase in ROS and 8-Iso-PGF2α levels, (4) reduced glutathione depletion, (5) protection against cholesterol-induced endoplasmic reticulum stress, (6) reversed cholesterol-induced decrease in A_1_ receptor levels	[201]
C57BL/6NCrl mice	chronically (twice weekly for 8 weeks) caffeine 5 mg/kg or 20 mg/kg (i.p.), followed 10 min later 10 mg/kg PQ first and 30 mg/kg MB second	Chronic dual-pesticide exposure model of Parkinson’s disease	Horizontal locomotor activity test	Caffeine at 20 mg/kg reduced TH+ neuron loss	[212]
Wistar rats	20 mg/kg (i.p.) 1 h before surgery and twice a day (10 mg/kg, i.p.) for 1 month; apomorphine hydrochloride (0.5 mg/kg, i.p.) 1 week before (baseline) and 4 weeks after the surgery with 1-day interval after the last caffeine injection	6-OHDA-induced neurotoxicity	Apomorphine-induced rotation tests	Caffeine (1) reduced apomorphine-induced rotations in a 6-OHDA toxicity model, (2) protected the neurons of substantia nigra pars compacta against 6-OHDA toxicity	[211]
Wistar rats	10 and 20 mg/kg (i.p.) daily for 14 days	6-OHDA-induced neurotoxicity	Apomorphine-induced rotation tests	Caffeine (1) reduced apomorphine-induced rotations in a 6-OHDA toxicity model, (2) reversed decreased noradrenaline and dopamine levels caused by 6-OHDA unilateral intrastriatal injection	[210]
Swiss Albino mice	20 mg/kg (i.p.) for 8 weeks	MPTP-induced neurotoxicity	–	Caffeine (1) partially protected MPTP-induced neurodegenerative changes, (2) modulated MPTP-mediated alterations in the expression and catalytic activity of CYP1A2, expression of adenosine A_2A_ receptor and DAT	[207]
Wistar rats	0.1, 0.3, or 1.0 mg/kg (i.p.) 45 min before the training session	MPTP-induced neurotoxicity	Two-way active avoidance test	Caffeine induced learning and memory improvement, what was independent of the locomotor stimulant effect; observed effects may be realized via dopamine/adenosine-receptor interaction	[206]
FVB mice	10 mg/kg/day (i.p.) for 2 weeks	MPTP-induced neurotoxicity	–	Caffeine (1) protected against loss of dopaminergic neuron in striatum, (2) attenuated gliosis, (3) blocked leakage of the blood–brain barrier in striatum, (3) blocked decreases in levels of striatal tight junction proteins, (4) blocked increases in MMP9 activity	[205]
C57BL6 mice	30 mg/kg (i.p.) for 8 days	MPTP-induced neurotoxicity	Paw grip strength test	Caffeine protected against (1) the reduction of paw grip strength, (2) perturbation in the homeostasis of neurometabolites in the striatum and olfactory bulb	[204]
C57BL6 mice	10, 20, 40 mg/kg (i.p.)	MPTP-induced neurotoxicity	–	Caffeine (1) produced a dose-dependent attenuation of MPTP-induced striatal dopamine loss in both young and retired breeder male, but not female, mice; (2) was less potent or altogether ineffective in female mice as a neuroprotectant after sham surgery compared to ovariectomy or after ovariectomy plus estrogen replacement compared to ovariectomy plus placebo treatment; (3) protection against dopamine loss in young male mice was blocked by estrogen administration	[208]
C57BL6 mice	30 mg/kg (i.p.)	MPTP-induced neurotoxicity	–	Caffeine (1) pre-treatment attenuated MPTP-induced striatal dopamine depletion when it was given 10 min, 30 min, 1 h, or 2 h but not 6 h before MPTP treatment; (2) post-treatment attenuated striatal dopamine loss when it was given 10 min, 30 min, 1 h or 2 h but not 4 h, 8 h or 24 h after MPTP injection; (3) metabolites also provide neuroprotective effect	[209]
Sprague–Dawley rats	1 g/l in drinking water	MPTP-induced neurotoxicity	–	Caffeine treatment (1) initiated simultaneously or during the course of ongoing neurodegeneration reduces loss of nigral dopaminergic neurons, (2) did not modify MPTP-induced decreases in striatal dopamine or tyrosine hydroxylase, (3) attenuated microglia activation in the substantia nigra but not in the striatum of MPTP-treated rats	[213]
Wistar rats	10 or 20 mg/kg/day in the drinking water	6-OHDA-induced neurotoxicity	Open field test, apomorphine-induced rotation tests	Caffeine treatment (1) blocked partially decreased locomotor activity and a high number of apomorphine-induced rotations, (2) increased dopamine contents and reversed the decrease dopamine level in the striatum, (3) improved the hippocampal neuronal viability, (4) increased TH+ in the striatum, (5) decreased the number of immunopositive cells for histone deacetylase and pro-inflammatory cytokines TNF-α and IL-1β in the 6-OHDA-lesioned group	[214]
Mongolian gerbils	0.1% caffeine drinking solution for 4 weeks	Ischemia model	–	Caffeine treatment (1) reduced the degree of ischemic necrosis of pyramidal cells of the CA1 hippocampal area after 5 min of bilateral carotid occlusion, (2) induced upregulation of A1 adenosine receptors in the CNS, what probably impaired the level of experimentally induced ischemic brain injury	[224]
Wistar rat pups	10 mg/kg (i.p.) immediately following HI induction	HI neonatal model	Water escape test, Morris water maze test	Caffeine treatment (1) attenuated deficits on the Morris water maze test observed in HI animals, (2) might be a potential therapeutic agent in reducing ischemic brain injury	[228]
Wistar rat pups	10 mg/kg/day (i.p.) immediately before HI and at 0, 24, 48 and 72 h post hypoxia	HI neonatal model	–	Caffeine treatment (1) reduced neuronal apoptosis in the developing brain, (2) might be effective in reducing ischemic brain injury	[229]
Wistar rat pups	10 mg/kg (i.p.) immediately after the 120 min of HI and 24 h following the initial injection	HI neonatal model	Rota rod test, silent gap detection, non-spatial water maze test	Caffeine treatment (1) significantly improved some behavioral outcomes in rat with a neonatal HI brain injury induced on postnatal day 6 and (2) partially rescued neuropathology	[230]
Sprague-Dawley rat	10 mg/kg (i.v.) 30 min prior to the induction of ischemia (acute treatment) 20 mg/kg (p.o.) three times daily per dose for the first week and 30 mg/kg (p.o.) three times daily for the second and third weeks; caffeine was withdrawn 24 h prior to ischemia. (chronic treatment)	Reversible forebrain ischemia model	–	Acute caffeine treatment (1) accelerated changes in the magnetic resonance images with increased hippocampal intensity appearing at 24 h post-ischemia, but (2) caused no changes in the extent of neuronal injury in any brain region compared to control-ischemic rats; (3) chronic caffeine treatment caused significantly less neuronal injury	[227]
Long-Evans rats	10 mg/kg of caffeine and 5% or 10% ethanol (0.325 or 0.65 g/kg, respectively) acute or chronic (3 weeks) (p.o.)	Carotid/middle cerebral artery occlusion model of ischemia	–	Caffeine plus ethanol treatment (1) almost entirely eliminated the ischemic injury, (2) initiated at 30-, 60-, 90-, and 120-min post-ischemia significantly reduced the infarct volume; (3) for 3 weeks prior to ischemia eliminates the neuroprotection seen after acute treatment	[273]
Long-Evans rats	2.5 h infusion at doses ranging from 2 to 10 mg/kg for caffeine and from 0.2 to 0.65 g/kg for ethanol	Carotid/middle cerebral artery occlusion model of ischemia	Sensorimotor tests: measurement of forelimb placing and foot-fault asymmetry	Caffeinol (0.2 g/kg of ethanol and 6 mg/kg of caffeine) treatment (1) reduced cortical infarct volume and (2) decreased behavioral dysfunction after transient carotid/middle cerebral artery occlusion	[274]
Sprague–Dawley rats	10 mg/kg caffeine and/or ethanol 0.32 g/kg infusion via the left femoral vein	Carotid/middle cerebral artery occlusion model of ischemia	Sensorimotor tests: measurement of forelimb placing and foot-fault asymmetry, postural reflex	Caffeinol treatment reduced size of excitotoxic lesion and caffeine may augmented the anti-ischemic effect of NMDA receptor blockers	[277]

**Table 4:** Summary of in vivo studies on the neuroprotective effects of chlorogenic acid.

Animals	Treatment	Model	Behavioral Tests	Main Outcomes	Ref.
Wistar rats	100 mg/kg (i.p.) for 24 days	Methotrexate-induced cerebellar Purkinje cell damage	–	(1) Reduced Purkinje cell damage and the expression of apoptotic cells, (2) decreased production of MDA and increase in SOD and catalase activity and GSH content in the cerebellum	[310]
Wistar rats	60 mg/kg (p.o.) for 30 days	Cadmium-induced brain damage	–	(1) Restored AChE, SOD, catalase, GSH-Px, and GST activity; (2) restored GSH, vitamins C and E, and lipid peroxidation level; (3) increased membrane-bound ATPase activity; (4) attenuated mitochondrial dysfunction and DNA fragmentation	[311]
ICR mice	3–9 mg/kg (p.o.) 30 min before scopolamine injection	Scopolamine-induced amnesia	Y-maze test, passive avoidance test, Morris water maze test	(1) Attenuation of the scopolamine-induced learning and memory impairment, (2) decreased AChE activity and MDA level in the hippocampus and frontal cortex.	[302]
Swiss Albino mice	1–10 mg/kg (p.o.) for 8 days before scopolamine injection	Scopolamine-induced amnesia	Y-maze test, novel object recognition test	(1) Attenuation of the scopolamine-induced learning and memory impairments, (2) decreased AChE and BChE activities in the cortex and hippocampus, (3) increased free radical scavenging activity	[304]
Wistar rats (5 days old pups)	100 and 200 mg/kg (p.o.) from PD 6 to 28 (with ethanol)	Alcohol-induced brain damage	Morris water maze test	(1) Attenuation of the altered cognitive function in ethanol-exposed pups, decreased AChE and caspase-3 activity, (2) reduced MDA and nitrite levels, (3) increased SOD and catalase activity, (4) decreased TNF-α and IL-1β levels, as well as decreased level of p65 of NF-κB in the cerebral cortex and hippocampus	[312]
C57BL/6 mice	100 mg/kg (i.p.) for 5 days	3-Nitropropionic acid induced neurotoxicity	–	Reduction of the 3-nitropropionic acid induced toxicity and genotoxicity	[313]
Wistar rats	15–60 mg/kg (p.o.) for 7 days before ischemia induction	Focal cerebral ischemia/reperfusion injury	Neurological deficit scoring	(1) Reduced mortality and improved neurological deficit scores, (2) decreased cerebral infarction area, (3) reduced ICAM-1 and VCAM-1 levels, (4) increased erythropoietin and HIF-1α levels, and (5) increased expression of NGF in the brain	[314]
Sprague-Dawley rats	20–500 mg/kg (p.o) for 7 days before ischemia induction	Cerebral ischemia/reperfusion injury	Neurological deficit scoring, step-down test, Y maze test	(1) Attenuation of the learning and memory impairments; (2) improved neurological deficit scores; (3) decreased cerebral infarction volume, cerebral water content and cerebral index; (4) promoted BDNF and NGF expression; (5) increased SOD activity and GSH levels; (6) decreased production of ROS, LDH, and MDA; (7) inhibited expression of caspase 3 and 9; and (8) promoted Nrf2, NQO-1, and HO-1 expression	[315]
Sprague-Dawley rats	3–30 mg/kg (i.p.) twice at 0 h and 2 h after ischemia induction	Focal cerebral ischemia/reperfusion injury	Balance-beam test	(1) Reduced sensory-motor functional deficits, infarct volume, BBB damage, and brain edema and (2) decreased lipid peroxidation and the expressions of matrix metalloproteinases	[316]
Charles foster albino rats	10 mg/kg (i.n.) after 2 h of occlusion	Global cerebral ischemia/reperfusion injury	–	(1) Reduced cerebral infarction volume and BBB damage; (2) restored the brain water content; (3) reduced calcium, nitrate, and glutamate levels in the cortex, hippocampus, cerebellum, and cerebrospinal fluid, and (4) decreased expression of TNF-α, iNOS, and caspase-3	[317]
Mongolian gerbils	100 µg/kg (i.p.) 60 min before injection of PEP-1-rpS3	Transient cerebral ischemia/reperfusion injury	–	Enhanced neuroprotective activity of PEP-1-rpS3 against the ischemia-induced hippocampal damage	[318]
Wistar rats	15–60 mg/kg (i.p.) 30 min after ischemia induction	Transient global ischemia/reperfusion injury	Morris water maze test	(1) Attenuation of the spatial memory impairment; (2) decreased CA1 pyramidal cell loss; (3) increased Bcl-2, SOD2, and CD31 expressions; and (4) decreased endothelin-1 expression	[319]
Mongolian gerbils	7.5–30 mg/kg (i.p.) for 5 days before ischemia induction	Transient global cerebral ischemia injury	8 Arm radial maze test, passive avoidance task	(1) Attenuation of cognitive impairment; (2) decreased CA1 pyramidal cell loss; (3) increased SOD2 expression; (4) reduced production of ROS, TNF-α, and IL-2 and elevated expression of IL-4 and IL-13	[320]
Sprague-Dawley rats	20–60 mg/kg (i.p.) 60 min before 6-OHDA injection, for 7 days	6-OHDA-induced neurotoxicity	Rotarod test, apomorphine-induced rotational test	(1) Reversed motor deficits, (2) attenuated decrease in striatal dopamine concentration, (3) reduced α-synuclein accumulation, (4) increased SOD and GSH-Px activities, and (5) restored Bcl-2/Bax expression in the striatum	[305]
C57BL/6J mice	50 mg/kg (p.o.) for 1 week before rotenone exposure, and then 5 days/week during the 4 weeks of rotenone treatment	Rotenone-induced neurotoxicity	–	(1) Prevented degeneration of dopaminergic neurons in the substantia nigra, (2) upregulated metallothionein-1 and 2 in striatal astrocytes	[321]
Swiss Albino mice	50 mg/kg (p.o.) for 24 days	MPTP-induced neurotoxicity	Rotarod test, pole test, traction test, catalepsy test	(1) Improved motor coordination and neurobehavioral activity; (2) improved mitochondria function; (3) reduced ROS generation; (4) increased SOD and mitochondrial GSH activity; (5) inhibited activation of proapoptotic proteins (Bax and caspase-3); (6) elevated expression of Bcl-2; (7) improved phosphorylation state of Akt, ERK1/2, and GSK3*β*	[322]
C57BL/6J mice	100 mg/kg (i.p.) for 7 days before LPS injection	LPS-induced neurotoxicity	–	Attenuation of the LPS-induced IL-1β and TNF-α release in the substantia nigra	[323]
Kunming mice	1 ml (p.o.) twice daily for 35 days	Kainic acid-induced neurotoxicity	Y maze test	(1) Attenuation of learning and memory impairment, (2) increased number of nNOS-positive neurons in the hippocampal CA1–4 regions	[324]
Swiss Albino mice	5 mg/kg (p.o.) for 15 days, last injection 30 min before pilocarpine	Pilocarpine-induced seizures	Seizure assessment (duration of clonic and tonic seizure)	(1) Anticonvulsant-like effect; (2) attenuated neuronal loss in the hippocampal CA1 region; (3) restored glutamate and GABA levels; (4) decreased NMDA, mGluR1, and mGluR5 receptor expression; (5) decreased lipid peroxidation and nitrite content; (6) increased SOD, catalase, and GSH activity; (7) restored AChE and monoamine oxidase activity	[325]
APP/PS2 transgenic mice	40 mg/kg (p.o.) for 180 days	Genetic model of Alzheimer’s disease	Morris water maze test	(1) Improved spatial memory, (2) decreased neuronal damage in the hippocampus, (3) inhibited autophagy, and (4) activation of the mTOR/TFEB signaling pathway	[299]

**Table 5:** Summary of in vivo studies on the neuroprotective effects of caffeic acid.

Animals	Treatment	Model	Behavioral Tests	Main Outcomes	Ref.
Mice (KM strain)	10 and 30 mg/kg (p.o.) 30 min before aluminum injection and then for 10 consecutive days	Aluminum-induced neurotoxicity	Passive avoidance task, water maze test	(1) Attenuation of the aluminum-induced impairment of learning and memory, (2) decreased MDA level, (3) increased choline acetyltransferase expression, (4) decreased expression of amyloid precursor protein of Aβ, and 5-LOX	[349]
Male Wistar rats	100 mg/kg (p.o.) for 11 days	Aluminum-induced neurotoxicity	Morris water maze test	(1) Improved memory; (2) reduced AChE, catalase, and GST activity; (3) reduced GSH and nitrite levels	[350]
Wistar rats	10–40 mg/kg (p.o.) for 21 days	Streptozotocin- induced dementia	Object recognition test, Morris water maze test, locomotor activity test	(1) Attenuation of the streptozotocin -induced learning and memory impairments; (2) increase in AChE activity; (3) increase in MDA, nitrite, and protein carbonyl levels; and (4) decrease in GSH level	[351]
Sprague–Dawley rats	100 mg/kg (i.p.) for 2 weeks	Aβ_1–40_-induced neurotoxicity	Morris water maze test	(1) Improved cognitive deficits, (2) decreased AChE activity and nitrite generation, (3) increased activity of catalase and GSH, (4) reduced IL-6 and TNF-α levels, (5) decreased NF-κB-p65 protein expression and caspase-3 activity, and (6) decreased p53 and p-p38 MAPK protein expression	[352]
Wistar rats	10–100 mg/kg (p.o.) for 30 days	–	Step-down inhibitory avoidance test, open field test	(1) Improved learning and memory; (2) decreased AChE activity in the cerebral cortex and striatum; and (3) increased AChE activity in the cerebellum, hippocampus, hypothalamus, and pons	[353]
Wistar rats	4 mg/kg (i.p.) 30 min before pilocarpine injection	Pilocarpine-induced seizures	Seizure assessment (latency to the first seizure, % seizures)	(1) Anticonvulsant-like effect, (2) decreased lipid peroxidation level and nitrite content, (3) increased SOD and catalase activity	[355]
Wistar rats	20 mg/kg (i.p.) for 5 days before quinolinic acid administration	Quinolinic acid-induced neurotoxicity	Circling behavior test, cylinder test	Attenuation of the quinolinic acid-induced behavioral alterations	[344]
Male Wistar rats	5 and 10 mg/kg (p.o.) for 21 days	Quinolinic acid-induced neurotoxicity	Locomotor activity test, rotarod test	(1) Improvement of locomotor activity and motor coordination, (2) restored redox status in striatum	[356]
Fisher rats	50 mg/kg (i.p.) 4 injections	Kainic acid-induced neurotoxicity	Seizure assessment (latency to seizures, seizure severity)	(1) Prolonged latency to seizures, (2) reduced neuronal loss in the CA3 hippocampal field	[357]
CF1 mice	4 and 8 mg/kg (i.p.) 30 min before seizure induction	Pilocarpine- and pentylenetetrazole-induced seizures	Seizure assessment (latency to the first seizure, % seizures)	(1) No anticonvulsant-like effect, (2) protection against pilocarpine-induced genotoxic damage in the hippocampus	[358]
CF1 mice	1–8 mg/kg (i.p.) 30 min before pentylenetetrazole injection, once every three day, for a total of 6 injections	Pentylenetetrazole -induced kindling	Seizure assessment (latency to the first seizure and the occurrence of clonic forelimb seizures)	(1) No antiepileptogenic-like effect, (2) protection against kindling-induced genotoxic damage in cerebral cortex, (3) decreased ROS production	[359]
Mongolian gerbils	10 and 20 mg/kg (p.o.) for 3 days before ischemia induction	Transient cerebral ischemia injury		(1) Decreased cell damage in the ischemic hippocampal CA1 region, (2) inhibition of microglia activation	[360]
Swiss mice	2–60 mg/kg (i.p.) for 5 days	Focal cerebral ischemia injury	Neurological deficit scoring, passive avoidance test, Y-maze test, water maze test, open field test	(1) Reduced infarcted area and improved neurological deficit scores, (2) improvement of working, spatial, and long-term aversive memory deficits, (3) attenuation of the ischemia-induced reduction in synaptophysin expression, and (4) increase in caspase 3 expression	[361]
Sprague–Dawley rats	50 mg/kg (i.p.) immediately after ischemia induction and then repeatedly for 12 h	Cerebral ischemia/reperfusion injury	Neurological deficit scoring	(1) Improved neurological deficit scores, (2) reduced infraction volume, (3) decreased 5-LOX expression	[362]
Sprague-Dawley rats	50 mg/kg (i.p.) 30 min before ischemia induction and 0, 1, 2 h after reperfusion in 1st day, and twice daily in the 2nd to 5th day	Focal cerebral ischemia/reperfusion injury	Neurological deficit scoring, inclined board test	(1) Reduction of neurological deficits, (2) decreased neuron loss, infarct volume, brain atrophy, and astrocyte proliferation, (3) inhibition of leukotriene production	[363]
Sprague–Dawley rats	10–50 mg/kg (i.p.) 30 min before ischemia induction	Global cerebral ischemia-reperfusion injury	Morris water maze test	(1) Attenuation of the ischemia-induced spatial learning and memory deficits, (2) reduced hippocampal neurons injury, (3) decreased MDA level, (4) increased SOD activity, and (5) suppressed 5-LOX overexpression	[364]
ICR mice	10 and 50 mg/kg (i.p.) 30 min, 2 and 6 h after cryoinjury on the 1st day and twice daily on days 2 to 7	Brain cryoinjury	–	(1) Reduced astrocyte proliferation and glial scar wall formation, (2) decreased expression of GFAP protein, (3) decreased SOD activity and (4) increased MDA level	[365]
Sprague-Dawley rats	50 mg/kg (p.o.) 10.5, 5.5, and 0.5 h before LPS injection	LPS-induced neurotoxicity	–	Attenuation of the LPS-induced loss of dopaminergic neurons and microglial activation in the substantia nigra	[367]
C57BL/6 mice	0.5–2% in diet, for 4 weeks	MPTP-induced neurotoxicity	_	(1) Decreased inflammatory cytokines levels; (2) suppressed NO, prostaglandin E2, and GFAP production; (3) reserved BDNF, GDNF, and tyrosine hydroxylase levels; (4) improved synthesis of dopamine	[370]
C57BL/6J mice	50 mg/kg (p.o.) for 1 week before rotenone exposure, and then 5 days/week during the 4 weeks of rotenone treatment	Rotenone-induced neurotoxicity	–	(1) Prevented degeneration of dopaminergic neurons in the substantia nigra, (2) upregulated metallothionein-1 and 2 in striatal astrocytes	[321]

**Table 6:** Summary of in vivo studies on the neuroprotective effects of trigonelline

Animals	Treatment	Model	Behavioral Tests	Main Outcomes	Ref.
ddY mice	500 mg/kg (p.o.) for 15 days	Aβ_25–35_-induced memory impairment	Morris water maze test	Attenuated memory impairment	[393]
Wistar rats	100 mg/kg (p.o.) for 3 days	Aβ_25–35_ induced neurotoxicity	Y maze test, novel object recognition task	(1) Attenuated learning and memory impairment; (2) alleviated hippocampal neuronal loss; (3) improved mitochondrial membrane potential; (4) restored MDA, protein carbonyl, and GSH levels; (5) reduced SOD and LDH activity; (6) reduced GFAP, S100b, COX-2, TNF-α, and IL-6 level in the hippocampus	[394]
Swiss Albino mice	50 and 100 mg/kg (p.o.) for 28 days	LPS-induced neurotoxicity	Morris water maze test, Y maze test	(1) Attenuated learning and memory disturbances, (2) decreased AChE activity, (3) restored SOD activity, (4) restored GSH and lipid peroxidation levels, (5) decreased TNF-α and IL-6 levels, and (6) increased BDNF level	[395]
Wistar rats	20–80 mg/kg (p.o.) for 7 days	LPS-induced neurotoxicity	Y maze test, Novel object discrimination test, passive avoidance test	(1) Attenuated learning and memory disturbances; (2) decreased MDA level and AChE activity; (3) increased SOD and catalase activity; (4) reduced GSH level; and (5) decreased NF-κB, TLR4, and TNF-α levels	[396]
Swiss Albino mice	20–80 mg/kg (p.o.) for 6 weeks	d-Galactose induced cognitive impairment	Morris water maze test, Y maze test	(1) Attenuated learning and memory disturbances, (2) decreased AChE activity, (3) decreased AGEs and MDA levels, (4) increased SOD activity and GSH level	[397]
Wistar rats	50 and 100 mg/kg (i.p.) for 3 days	6-OHDA-induced neurotoxicity	Apomorphine-induced rotation test	(1) Reduced rotational behavior, (2) increased viability of neurons in substantia nigra, (3) prevented apoptosis, (4) reduced MDA and nitrite levels, and (5) increased GSH level	[398]
Wistar rats	*Trigonella foenum-graecum* extract (82% trigonelline) 30–100 mg/kg (p.o.), 2 weeks after 6-OHDA injection	6-OHDA-induced neurotoxicity	Apomorphine-induced rotation test	Increased number of ipsilateral rotations	[399]
C57BL/6 mice	*Trigonella foenum-graecum* extract (82% trigonelline) 30 mg/kg (p.o.), 60 min before or after MPTP	MPTP-induced neurotoxicity	Open field test	Improved spontaneous locomotor activity in the pre-treatment schedule	[399]
Sprague–Dawley rats	25–100 mg/kg (i.p.) twice (30 min before and immediately after ischemia induction)	Cerebral ischemia/reperfusion injury	Neurological deficit scoring, rotarod test	(1) Improved motor coordination and neurodeficit scores, (2) decreased cerebral infarction volume, (3) reduced nitrite and MDA levels, (4) increased GSH level, and (5) decreased expression of myeloperoxidase	[400]

## Abbreviations

Aβ	Amyloid beta
AChE	Acetylcholinesterase
AGEs	Advanced glycation end products
Akt	Protein kinase B
APP	Amyloid precursor protein
APPsw	Swedish mutation mice, mice carrying the mutant APPK670N, M671L gene
ATP	Adenosine-5′-triphosphate
Bax	Bcl-2-associated X protein
BBB	Blood brain barrier
BChE	Butyrylcholinesterase
Bcl2	B-cell lymphoma protein 2
BDNF	Brain-derived neurotrophic factor
CAPE	Caffeic acid phenyl ester
CBF	Cerebral blood flow
CD31	Platelet/endothelial cell adhesion molecule-1
CNS	Central nervous system
COX-2	Cyclooxygenase 2
CSF	Cerebrospinal fluid
CYP	Cytochrome P450
DAT	Dopamine transporter
ER	Endoplasmic reticulum
ERK1/2	Extracellular signal-regulated kinase-1 and -2
GABA	Gamma-aminobutyric acid
GDNF	Glial cell line-derived neurotrophic factor
GFAP	Glial fibrillary acidic protein
GSK3β	Glycogen synthase kinase 3 beta
GSH	Reduced glutathione
GSH-Px	Glutathione peroxidase
GST	Glutathione-S-transferase
HI	Hypoxia-ischemia
HIF1α	Hypoxia-inducible factor 1 alpha
HO-1	Heme oxygenase 1
ICAM-1	Intercellular adhesion molecule 1
IL-1β	Interleukin 1 beta
IL-2	Interleukin 2
IL-4	Interleukin 4
IL-6	Interleukin 6
IL-13	Interleukin 13
i.n.	Intranasal
iNOS	Inducible nitric oxide synthase
i.p.	Intraperitoneally
i.v.	Intravenously
LDH	Lactate dehydrogenase
5-LOX	5-Lipoxygenase
LPS	Lipopolysaccharide
MAPK	Mitogen-activated protein kinase
MB	Manganese bisethylenedithiocarbamate
MDA	Malondialdehyde
mGluR1	Metabotropic glutamate receptor type 1
mGluR5	Metabotropic glutamate receptor type 5
MMP-2, -9	Metallomatrixprotease-2,-9
MPTP	1-Methyl-4-phenyl-1,2,3,6-tetrahydropyridine
MRI	Magnetic resonance imaging
mTOR	Mammalian target of rapamycin
NF-κB	Nuclear factor kappa-light-chain-enhancer of activated B cells
NGF	Nerve growth factor
NMDA	*N*-methyl-d-aspartate
nNOS	Neuronal nitric oxide synthase
NO	Nitric oxide
NOS-2	Nitric oxide synthase-2
NQO-1	NAD(P)H quinone oxidoreductase
Nrf2	Nuclear factor erythroid 2-related factor 2
6-OHDA	6-Hydroxydopamine
p53	Tumor protein p53
p65	Transcription factor p65
PARP-1	Poly [ADP-ribose] polymerase 1
p-JNK	C-Jun *N*-terminal kinases
p.o.	Orally
PQ	1,1′-Dimethyl-4,4′-bipyridinium dichloride hydrate
RNS	Nitrogen reactive species
ROS	Reactive oxygen species
rpS3	Ribosomal protein
S100b	S100 calcium-binding protein B
SNP	Sodium nitroprusside
SOD	Superoxide dismutase
SOD2	Superoxide dismutase 2
TFEB	Transcription factor EB
TH+	Tyrosine hydroxylase immunoreactivity
TLR4	Toll-like receptor 4
TNF-α	Tumor necrosis factor α
TrkB	Tirosine kinase receptor
UDP	Uridine 5′-diphosphate
VCAM-1	Vascular cell adhesion protein 1
